# Extracellular Enolase-1 Promotes CAF-Associated Stromal Reprogramming via the Plasmin/TGF-β Axis in Multiple Myeloma

**DOI:** 10.3390/cancers18091467

**Published:** 2026-05-02

**Authors:** I-Che Chung, Tung-Yueh Chuang, Yu-Tung Ko, Mao-Lin Chen, Po-Yang Hsu, Wei-Ching Huang, Ta-Tung Yuan

**Affiliations:** Department of Research and Development, HuniLife Biotechnology, Inc., Taipei 114, Taiwan; icchung@hunilife.com (I.-C.C.); benz8821001@gmail.com (T.-Y.C.); ytko@hunilife.com (Y.-T.K.); zato9198@gmail.com (M.-L.C.); superyang@hunilife.com (P.-Y.H.); wchuang@hunilife.com (W.-C.H.)

**Keywords:** extracellular enolase-1, tumor microenvironment, transforming growth factor-beta, monoclonal antibody, cancer-associated fibroblast, multiple myeloma

## Abstract

**Background**: Stromal remodeling in the tumor microenvironment contributes to multiple myeloma (MM) progression and drug resistance, but the extracellular mediators that drive these changes remain incompletely defined. Extracellular enolase-1 (ENO1), including membrane-associated and secreted forms, has been implicated in tumor progression; however, its role in cancer-associated fibroblast (CAF)-associated stromal reprogramming in MM is unclear. **Methods**: The effects of extracellular ENO1 on stromal activation and tumor-supportive functions were examined in MM using MM–bone marrow stromal cell (BMSC) co-cultures, lactate production and viability assays, immunoblotting, cytokine analyses, and a subcutaneous xenograft model of bortezomib (BTZ)-resistant MM in male 6–7-week-old NOD.Cg-Prkdc^scid^ Il2rg^tm1Vst^/Vst (NPG) mice. HuL001, an anti-ENO1 monoclonal antibody, was used to evaluate the therapeutic relevance of extracellular ENO1 targeting. **Results**: Extracellular ENO1 promoted fibroblast activation protein expression through plasmin-mediated transforming growth factor-β (TGF-β) activation and induced a CAF-associated stromal phenotype marked by enhanced glycolytic activity and increased secretion of tumor-promoting cytokines in MM-BMSC co-cultures. HuL001 suppressed these ENO1-driven effects. HuL001-pretreated stromal cells also exhibited reduced tumor-supportive activity in a BTZ-resistant MM xenograft model. In addition, HuL001 combined with lenalidomide overcame BTZ resistance in MM. **Conclusions**: Extracellular ENO1 drives CAF-associated stromal reprogramming in the MM microenvironment through the ENO1/plasminogen/plasmin/TGF-β axis. Therapeutic targeting of extracellular ENO1 with HuL001 may disrupt these tumor-supportive stromal activities and help overcome drug resistance in MM.

## Simple Summary

Multiple myeloma is influenced not only by cancer cells themselves but also by surrounding stromal cells in the bone marrow. This study investigated whether extracellular enolase-1, a protein present on the cell surface or released by myeloma cells, contributes to stromal changes that favor tumor growth and drug resistance. The results showed that extracellular enolase-1 promoted a tumor-supportive stromal state through plasmin-dependent activation of transforming growth factor beta, accompanied by increased production of factors linked to cancer progression. Blocking extracellular enolase-1 with the antibody HuL001 reduced these stromal changes and improved the antitumor effect of lenalidomide in bortezomib-resistant multiple myeloma models. These findings suggest that extracellular enolase-1 may represent a potential therapeutic target for disrupting harmful interactions between myeloma cells and the bone marrow microenvironment.

## 1. Introduction

Multiple myeloma (MM) is a malignancy of terminally differentiated plasma cells originating in the bone marrow. MM represents one of the most common hematologic malignancies worldwide. The treatment of MM has notably advanced with the approval of novel agents [1], including (i) proteasome inhibitors such as bortezomib (BTZ), carfilzomib and ixazomib; (ii) immunomodulatory drugs such as lenalidomide (LEN) and pomalidomide; (iii) monoclonal antibodies (mAbs) such as daratumumab and elotuzumab; (iv) antibody-drug conjugates (ADCs); (v) chimeric antigen receptor T-cell (CAR-T) cell therapy; and (vi) emerging treatments such as bispecific antibodies. However, MM cells frequently develop drug resistance through alterations in cellular signaling pathways. Continued research into new therapeutic targets and combination strategies is crucial for patients with relapsed or refractory multiple myeloma (RRMM).

Cancer-associated fibroblasts (CAFs) are key components of the tumor microenvironment (TME), and play crucial roles in the progression [2], invasion [3] and therapeutic resistance [4] of cancer. CAFs originate from normal fibroblasts and undergo differentiation through signaling interactions with cancer cells, resulting in their activation [5] and phenotypic changes [6]. Transforming growth factor (TGF)-β plays a crucial role in the activation of CAFs, promoting their differentiation and secretion of extracellular matrix components that support tumor progression [7]. Activated CAFs contribute to MM progression by enhancing glycolysis, promoting immune evasion and inducing drug resistance. These effects are mediated in part through the secretion of cytokines such as interleukin (IL)-6, growth factors such as vascular endothelial growth factor (VEGF) and metabolic intermediates such as lactate [8,9,10,11]. CAFs proliferating under hypoxic conditions exhibit enhanced glucose uptake and utilization. This metabolic shift increases lactate production and promotes lactate accumulation in the TME, which acidifies the microenvironment, promoting angiogenesis through VEGF, facilitating cancer cell adaptation to hypoxia, suppressing immune cell function and fostering metastasis [12]. Fibroblast activation protein (FAP) is primarily expressed by activated fibroblasts, particularly CAFs, in the TME [13]. FAP can be induced by TGF-β, and has emerged as a promising target for cellular immunotherapies. FAP-specific CAR-T cells are currently being explored to target bone marrow (BM)-CAFs to overcome CAF-induced CAR-T-cell inhibition in MM treatment [9].

Enolase-1 (ENO1, also known as α-enolase) is a glycolytic enzyme that catalyzes the conversion of 2-phospho-D-glycerate to phosphoenolpyruvate in the cytosol [14,15]. Beyond its metabolic role, ENO1 can be translocated to the cell surface, where it acts as a plasminogen receptor to facilitate plasmin activation [16]. It has been shown that ENO1 promotes Burkitt lymphoma progression via plasmin-mediated TGF-β activation [17]. Clinically, ENO1 overexpression has been reported across multiple malignancies, including MM, and is associated with poor prognosis. In MM, higher ENO1 levels correlate with worse overall survival [18,19]. Our previous study on MM demonstrated that extracellular ENO1 enhanced glycolytic activity and oncogenic behaviors in MM cells, including cell migration, survival and secretion of tumor-promoting factors [20]. Similarly, a previous study showed that exosome-derived ENO1 enhanced tumor cell motility in hepatocellular carcinoma (HCC) by regulating integrin α6β4 expression [21]. Our previous study also demonstrated that HuL001 reduced tumor growth in prostate cancer (PCa) xenografts by modulating the TME [22]. Furthermore, extracellular ENO1 within the TME was implicated in promoting PCa cell migration and metastasis [23]. Additional evidence suggested that ENO1 is upregulated when MM cells are co-cultured with plasmacytoid dendritic cells (pDCs). Pharmacological ENO1 inhibition enhanced pDC-triggered T- and natural killer cell-mediated anti-MM activity. Together, these findings support a role for ENO1 in promoting MM progression within the BM TME through interactions with multiple stromal and immune cell components [19]. Therefore, targeting ENO1 to disrupt metabolic reprogramming in the TME represents a promising therapeutic strategy for MM.

The present study investigated the potential roles of extracellular ENO1 in the TME, where CAFs, enhanced glycolysis, and tumor-promoting factors such as IL-6 and VEGF contribute to MM progression. The current study also examined whether extracellular ENO1 could affect CAF differentiation and secretion of tumor-promoting factors. A humanized ENO1-specific antibody (HuL001) was used to validate the role of extracellular ENO1 in CAF-mediated tumor growth in vivo. To evaluate the role of extracellular ENO1 in a human MM-relevant in vivo context, immunodeficient NPG mice were used to support stable engraftment of human MM cells and to enable assessment of tumor-stroma interactions. The possible mechanism of ENO1-driven reprogramming of the TME was investigated using in vitro co-culture systems of MM cells and BM stromal cells (BMSCs), together with cytokine and metabolic analyses, immunoblotting, and in vivo MM xenograft models. Furthermore, a novel therapeutic strategy was proposed to treat BTZ-resistant MM by targeting extracellular ENO1 to suppress the tumor-promoting activity of CAFs. To the best of our knowledge, the present study is the first one to demonstrate that extracellular ENO1 can regulate the differentiation and functionality of CAFs in the TME via plasmin-mediated TGF-β activation.

## 2. Materials and Methods

### 2.1. Cell Culture

The human MM cell lines KMS-11 (cat. no. JCRB1179) and KMS-11/BTZ (cat. no. JCRB1642) were obtained from the Japanese Collection of Research Bioresources Cell Bank (JCRB; Osaka, Japan) with short tandem repeat (STR)-PCR profiling performed at JCRB, while RPMI-8226 cells (cat. no. 60384) were obtained from the Bioresource Collection and Research Center (BCRC; Hsinchu City, Taiwan) and authenticated using STR profile analysis at BCRC. To generate BTZ-resistant MM cells, RPMI-8226/BTZ cells were established from parental RPMI-8226 cells through repeated cycles of 1-day BTZ treatment (starting at 2.5 nM and doubling to 5 nM, followed by 10 nM after recovery culture) followed by 1-day drug-free recovery. The immortalized human BMSCs HS-5 (cat. no. CRL-3611) were obtained from the American Type Culture Collection (ATCC; Manassas, VA, USA) and were authenticated using STR profile analysis at ATCC. Cells were cultured in RPMI-1640 medium (cat. no. 11875093; Gibco (Grand Island, NY, USA); Thermo Fisher Scientific, Inc. (Waltham, MA, USA)) supplemented with 10% fetal bovine serum (FBS; cat. no. 26140079, Gibco; Thermo Fisher Scientific, Inc.) and 50 U/mL penicillin-streptomycin (cat. no. 15140122, Gibco; Thermo Fisher Scientific, Inc.). Cells were maintained at 37 °C in a humidified atmosphere containing 5% CO_2_. According to the suppliers’ quality control documentation, the cell lines obtained from JCRB, BCRC, and ATCC were tested for mycoplasma contamination prior to distribution. KMS-11 and KMS-11/BTZ were used as the parental and BTZ-resistant MM models, respectively, for BTZ sensitivity testing and xenograft studies. HS-5 cells were used as the human BMSC model for direct co-culture with MM cells to generate MM-educated BMSCs. RPMI-8226 and RPMI-8226/BTZ were used as an additional parental/BTZ-resistant MM pair to evaluate whether the stromal response could be reproduced in an independent BTZ-resistant MM model.

### 2.2. MM Xenografts and MM-TME Mouse Models

All in vivo animal experiments were conducted at TFBS Bioscience, an external facility that provided support for the animal studies, and were performed according to protocols approved by the Institutional Animal Care and Use Committee (IACUC) of TFBS Bioscience (approval nos. TFBS2024-002 and TFBS2025-002).

Animals were acclimated for at least 7 days before initiating experiments. All animals were of specific pathogen-free (SPF) status with no prior experimental interventions. Housing conditions were maintained at a temperature of 20–24 °C, relative humidity between 40–60%, and a 12-h light/dark cycle. Each cage contained five animals of the same species. The sample size was chosen based on prior studies and anticipated effect size, without a formal calculation. Animals were randomly allocated using random number tables, and cage positions were regularly rotated to reduce environmental variability. A total of 69 male 6–7-week-old NOD.Cg-Prkdc^scid^ Il2rg^tm1Vst^/Vst (NPG) mice were used (Lasco Co., Ltd. ((Taipei, Taiwan))). For MM xenografts, KMS-11 and KMS-11/BTZ cells were washed with serum-free RPMI 1640 and resuspended in a 1:1 (*v*/*v*) mixture of serum-free RPMI 1640 and Matrigel (cat. no. 356231; Corning, Inc. (Corning, NY, USA)). KMS-11 cells (5 × 10^6^ cells/mouse) or KMS-11/BTZ cells (10 × 10^6^ cells/mouse) were implanted subcutaneously into the right flank of mice. When the mean tumor size reached 100 mm^3^ (set as day 0), the mice were randomized to the following treatment groups: (i) PBS (vehicle control, twice per week), (ii) HuL001 (30 mg/kg, twice per week), (iii) LEN (5 mg/kg, 5 consecutive days of treatment followed by 2 days of rest per week) or (iv) HuL001 plus LEN. All treatments were administered by intraperitoneal injection. Blinding was not implemented during outcome assessment. No animals were excluded from the analyses unless required by predefined humane endpoint criteria. The primary outcome of this study was tumor volume or percentage tumor growth inhibition (TGI) measured at the study endpoint, which was calculated using the formula [ΔT/ΔC × 100], where ΔT is the tumor volume difference between the vehicle group and the drug-treated group, and ΔC is the tumor volume difference between the vehicle group and the tumor’s initial volume [24]. For MM-TME mouse models, MM-educated BMSCs (also referred to as MM-associated CAF-like cells) were generated through direct co-culture of HS-5 BMSCs (a source of CAFs) with KMS-11/BTZ cells at a 1:2 ratio, with or without HuL001 (10 µg/mL) treatment for 5 days. For implantation, KMS-11/BTZ cells were mixed with or without MM-educated BMSCs and resuspended in serum-free RPMI 1640 supplemented 1:1 (*v*/*v*) with Matrigel. Subsequently, 10 × 10^6^ KMS-11/BTZ cells, with or without 1 × 10^6^ MM-educated BMSCs, were injected subcutaneously into the right flank of NPG mice. Humane endpoints were predefined as follows: (i) the maximum permitted tumor size was a mean tumor diameter of 20 mm [tumor volume = (shorter diameter^2^ × longer diameter)/2] and (ii) the maximum permitted tumor burden was 10% of the animal’s body weight. Animals were monitored at least once daily for general condition and signs of ulceration, necrosis, or distress, and tumor size was measured regularly throughout the study. Mice were euthanized when either limit was exceeded or earlier for ulceration/necrosis/distress. No expected or unexpected adverse events were observed during the study. At the end of the study, the animals were euthanized by CO_2_ inhalation (at a displacement rate of 30–70% of the cage volume per min), in compliance with IACUC protocols to minimize potential distress. Death was subsequently confirmed by absence of respiration and heartbeat, and lack of reflexes, before tissue collection. No publicly registered study protocol was prepared prior to study initiation. However, the study objectives and experimental procedures were defined in advance and conducted under approved IACUC protocols.

### 2.3. Measurement of MM Cell Viability After Co-Culturing with MM-Educated BMSCs

MM-educated BMSCs were generated by directly co-culturing HS-5 BMSCs with KMS-11/BTZ cells at a 1:2 ratio, with simultaneous treatment using 10 µg/mL HuL001 or 10 µg/mL control hIgG1 for 5 days. Co-culturing MM cells with MM-educated BMSCs was achieved by using Transwell inserts (Millicell hanging cell culture insert, polyethylene terephthalate 0.4 µm; cat. no. PTHT24H48; Merck KGaA (Darmstadt, Germany)), which were placed in 24-well culture plates. KMS-11/BTZ cells (2 × 10^4^) were seeded into the inserts, while MM-educated BMSCs (2 × 10^3^) were placed in the lower compartment of the culture system for 5 days. Subsequently, the KMS-11/BTZ cells were collected, and their viability was assessed by Cell Counting Kit-8 (CCK-8; cat. no. CK04-20; Dojindo Laboratories, Inc. (Kumamoto, Japan)) assay as per the manufacturer’s instructions. The assay is based on the reduction of WST-8 by cellular dehydrogenases in viable cells to generate a water-soluble formazan dye, which is quantified by measuring absorbance at 450 nm. The absorbance is proportional to the number of viable cells in the sample.

### 2.4. HuL001, Recombinant ENO1, and Other Reagents

Production of the proprietary HuL001 by HuniLife was conducted as described previously [22]. HuL001 was a humanized IgG1 antibody and cross-reactive to both human and mouse ENO1, but not ENO2 or ENO3. The anti-*Klebsiella pneumoniae* antibody (hIgG1 backbone; provided by Dr Shih-Chong Tsai, Development Center for Biotechnology, Taipei, Taiwan) was used as isotype control in the in vitro studies. The gene encoding human ENO1 protein was cloned into a pTrcHis vector, in which the expression of the transgene was isopropyl-β-D-thiogalactoside-inducible. Tranexamic acid (TXA; cat. no. T1810000; European Pharmacopoeia), recombinant human TGF-β1 (cat. no. 100-21; PeproTech, Inc. (Cranbury, NJ, USA)) and TGF-β receptor kinase inhibitor SB431542 (cat. no. S1067; Selleck Chemicals (Houston, TX, USA)) were also used in the present study.

### 2.5. IC_50_ Determination

Drug concentrations were log-transformed prior to analysis. OD450 values were normalized to the untreated control group, which was set as 100% cell viability. Dose–response curves were fitted by nonlinear regression using the log(inhibitor) vs. normalized response model in GraphPad Prism version 8.0.2 (GraphPad Software), and IC_50_ values were calculated from the fitted curves.

### 2.6. RNA Interference

Hexokinase 2 (HK2; cat. no. 1433; Thermo Fisher Scientific, Inc.) and hypoxia-inducible factor 1 (HIF)-1α (cat. no. AM51331; Invitrogen (Carlsbad, CA, USA); Thermo Fisher Scientific, Inc.) siRNA sequences were as follows: si-HK2 sense, 5′-GGUUG ACCAG UAUCU CUACT T-3′ and antisense, 5′-GUAGA GAUAC UGGUC AACCT T-3′; si-HIF1A sense, 5′-GGGUA AAGAA CAAAA CACA-3′ and antisense, 5′-UGUGU UUUGU UCUUU ACCC-3′; and scramble siRNA sense 5′-UUCUC CGAAC GUGUC ACGUT T-3′ and antisense, 5′-ACGUG ACACG UUCGG AGAAT T-3′). Cells were transfected with 50 nM dsRNA duplexes using RNAiMAX (cat. no. 13778; Invitrogen; Thermo Fisher Scientific, Inc.) according to the manufacturer’s instructions.

### 2.7. Flow Cytometry Detection of Cell Surface ENO1

KMS-11 and KMS-11/BTZ cells were incubated with Fc-block receptor (cat. no. 130-059901; Miltenyi Biotec, Inc. (Bergisch Gladbach, Germany)) for 10 min at 4 °C and then washed once with cold Stain Buffer (cat. no. 554656; BD Biosciences (San Jose, CA, USA)) at 300× *g* for 5 min. Cells were stained with anti-ENO1 antibody (1:50; cat. no. H00002023-M01; Abnova Corporation (Taipei, Taiwan)) or isotype-matched mouse IgG1 (1:50; cat. no. 401402; BioLegend, Inc., (San Diego, CA, USA)) for 30 min at 4 °C, and then washed twice in cold Stain Buffer at 300× *g* for 5 min. Cells were then incubated with PE-conjugated goat anti-mouse IgG (1:20; cat. no. 405307; BioLegend, Inc.) for 30 min at 4 °C, followed by washing twice in cold Stain Buffer at 300× *g* for 5 min. After staining, the samples were resuspended in cold Stain Buffer and analyzed using a CytoFlex flow cytometer (Beckman Coulter, Inc. (Brea, CA, USA)). Data were acquired using CytExpert 2.4 software (Beckman Coulter, Inc.) and analyzed using Kaluza Analysis 2.1 Software (Beckman Coulter, Inc.). For each sample, 1 × 10^4^ cells were acquired. The percentage of surface ENO1-positive cells was determined by subtracting the percentage of the samples from the isotype control.

### 2.8. Western Blotting

Cell lysates were prepared and subjected to immunoblotting analysis following standard protocols. Xenograft tumor tissues from each group were homogenized, and the total lysates were also subjected to immunoblotting analysis according to standard protocols. Briefly, tissues were homogenized under liquid nitrogen in a precooled mortar and ground into a fine powder using a pestle. The resulting powder was resuspended in PBS containing 1X Protease and Phosphatase Inhibitor Cocktail (cat. no. 78442; Thermo Fisher Scientific, Inc.), followed by centrifugation at 300× *g* for 2 min at room temperature. The supernatants were collected and sonicated in T-PER Tissue Protein Extraction Reagent (cat. no. 78510; Thermo Fisher Scientific, Inc.) supplemented with 1X Protease and Phosphatase Inhibitor Cocktail. The samples were then centrifuged at 12,000× *g* for 10 min at 4 °C. Protein concentrations were determined using a BCA Protein Assay Kit (cat. no. 23225; Pierce; Thermo Fisher Scientific, Inc.). The primary antibodies used were against ENO1 (1:2000; cat. no. ab190365; Abcam (Cambridge, UK)), FAP (1:2000; cat. no. ab207178; Abcam), HK2 (1:2000; cat. no. 2867; Cell Signaling Technology, Inc. (Danvers, MA, USA)), fibroblast-specific protein 1 (FSP1) (1:2000; cat. no. 16105-1-AP; Proteintech Group, Inc. (Rosemont, IL, USA), platelet-derived growth factor receptor (PDGFR)β (1:2000; cat. no. 3169; Cell Signaling Technology, Inc.), α-smooth muscle actin (SMA) (1:2000; cat. no. ab5694; Abcam), HIF-1α (1:1000; cat. no. IR113-466; iReal Biotechnology, Inc. (Hsinchu City, Taiwan)) and GAPDH (1:5000; cat. no. sc-32233; Santa Cruz Biotechnology, Inc. (Dallas, TX, USA)). The secondary antibodies included in the present study were HRP-conjugated anti-mouse antibody (1:5000; cat. no. 2076; Cell Signaling Technology, Inc.) and HRP-conjugated anti-rabbit antibody (1:5000; cat. no. 7074; Cell Signaling Technology, Inc.).

### 2.9. Detection of Secreted ENO1

The levels of secreted ENO1 in cell culture supernatants were measured using the Human ENO1 ELISA Kit (α-ENO) (cat. no. ab181417; Abcam) according to the manufacturer’s instructions.

### 2.10. Measurement of Lactate

The levels of lactate in the cell culture supernatant were measured using a colorimetric lactate assay kit (cat. no. MET-5012; Cell Biolabs (San Diego, CA, USA)) according to the manufacturer’s instructions.

### 2.11. Cytokine ELISA

Measurement of cytokine levels was performed using ELISA kits, including human IL-6 (cat. no. DY206-05; R&D Systems, Inc. (Minneapolis, MN, USA)), human VEGF (cat. no. DY293B-05; R&D Systems, Inc.) and human TGF-β1 (cat. no. DY240-05; R&D Systems, Inc.) according to the manufacturer’s instructions.

### 2.12. Measurement of Glucose Uptake

To evaluate glucose uptake, a colorimetric assay kit (cat. no. ab136955; Abcam) was used according to the manufacturer’s instructions. Briefly, KMS-11 and KMS-11/BTZ cells were cultured for 18 h. Cells were then washed twice with PBS and cultured in glucose-free RPMI (cat. no. 11879020; Gibco; Thermo Fisher Scientific, Inc.) for 1 h. Subsequently, the cells were incubated in PBS for 40 min and then with 1 mM 2-deoxy-D-glucose (2-DG) for 20 min. Next, cells were washed three times with PBS to remove exogenous 2-DG, lysed with extraction buffer, freeze/thawed once and heated at 85 °C for 40 min to degrade endogenous NAD(P), followed by centrifugation at 300× *g* for 2 min. The resulting supernatant was analyzed for 2-DG-6-phosphate content using a microplate reader at 412 nm. Cells treated with 100 μM phloretin were used as a positive control.

### 2.13. Measurement of the Plasminogen Receptor Activity of ENO1

HS-5 BMSCs were harvested, washed twice with PBS at 300× *g* for 2 min, and resuspended in PBS containing 50% conditioned medium (CM) derived from KMS-11/BTZ cells at a concentration of 1 × 10^6^ cells/mL. The cells were preincubated at 37 °C for 3 h with or without ENO1 protein (10 μg/mL), HuL001 (100 μg/mL) and hIgG1 (100 μg/mL). Subsequently, cells were treated with 120 nM human Glu-plasminogen (cat. no. 528180; Sigma-Aldrich; Merck KGaA) for 1 h, washed three times with PBS and resuspended in 100 μL PBS containing 120 nM tissue plasminogen activator (cat. no. 10157-HNCH2; Sino Biological (Beijing, China)) and 1 μM plasmin chromogenic substrate Chromogenix S-2251 (cat. no. S820332; DiaPharma (Kashihara City, Japan)) at 37 °C for 1–3 h. Plasminogen receptor activity (plasmin activation) was assessed by measuring the absorbance at 405 nm.

### 2.14. Statistical Analysis

Statistical analyses were performed with GraphPad Prism 8.0 (GraphPad; Dotmatics (Boston, MA, USA)). Data are presented as the mean ± standard deviation from independent in vitro experiments and as the mean ± standard error of the mean for biological replicates in in vivo studies, with the corresponding replicate numbers indicated in the figure legends. Data normality and variance homogeneity were routinely evaluated, and when these assumptions were not met, appropriate non-parametric tests were applied. Differences between two groups were analyzed using two-tailed unpaired Student’s *t*-tests. Comparisons of multiple groups were performed using one-way analysis of variance followed by Tukey’s post hoc test. Two-sided *p* < 0.05 was considered to indicate a statistically significant difference.

## 3. Results

### 3.1. Total, Surface and Secreted ENO1 Levels, and Glycolytic Activity Are Increased in BTZ Resistant MM Cells

**Figure 1:**
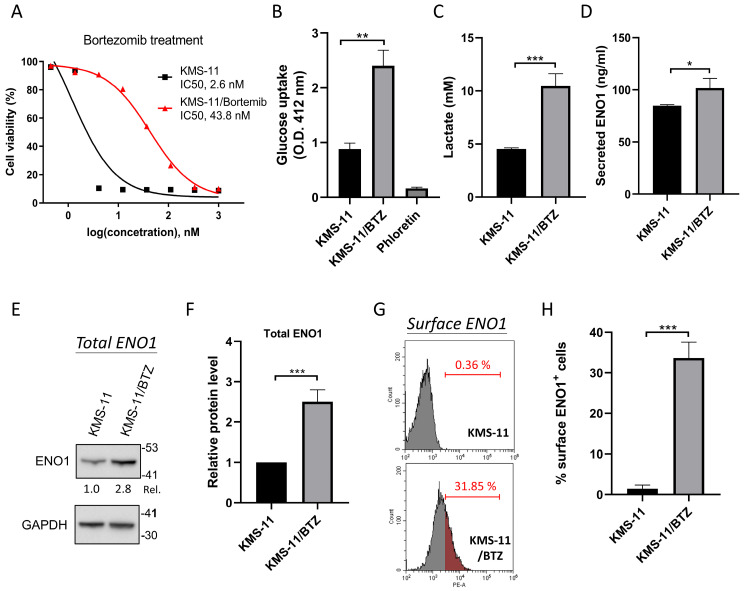
Total, surface and secreted ENO1 levels as well as glycolytic activity were increased in BTZ-resistant MM cells. (**A**) KMS-11 and KMS-11/BTZ (BTZ-resistant) MM cells were incubated with various doses of BTZ for 3 days. Inhibition of cell proliferation was measured by Cell Counting Kit-8 assay. (**B**–**H**) KMS-11 and KMS-11/BTZ cells were cultured for 18 h, and then the supernatant was collected to determine the levels of (**C**) lactate and (**D**) secreted ENO1. (**B**) Glucose uptake was analyzed. (**E**) Representative immunoblots of total ENO1 protein levels in cell lysates from one of three independent biological experiments, with GAPDH used as a loading control. (**F**) Densitometric quantification of total ENO1 immunoblot signals from the same experimental set, based on three independent biological experiments. (**G**) Representative flow cytometry histogram of cell-surface ENO1 expression from one of three independent biological experiments. (**H**) Quantitative analysis of cell surface ENO1 expression from the same experimental set, based on three independent biological experiments. Immunoblot signals were normalized to GAPDH and expressed as relative levels versus parental KMS-11 (set to 1.0). The results in panels (**B**–**D**,**F**,**H**) are presented as the mean ± standard deviation from three independent biological experiments. Statistical analysis was performed using two-sided unpaired Student’s *t*-test. * *p* < 0.05, ** *p* < 0.01, *** *p* < 0.001. Original Western blot images corresponding to the blots shown in this figure are provided in Figure S6.

BTZ sensitivity was first evaluated in parental and BTZ-resistant KMS-11 cells (Figure 1A). Parental KMS-11 cells exhibited high sensitivity to BTZ, with a half-maximal inhibitory concentration (IC_50_) of 2.6 nM. By contrast, BTZ-resistant KMS-11/BTZ cells displayed significant resistance to BTZ, with an IC_50_ of 43.8 nM, which is approximately 16-fold higher than that of parental cells. Previous studies have shown that BTZ resistance in MM cells is associated with upregulation of glycolysis [25,26,27]. This metabolic reprogramming of BTZ-resistant MM cells is characterized by increased glucose uptake and lactate secretion—the final step of glycolysis, so called the Warburg effect [28]. In agreement, the present data also confirmed the increase in both glucose uptake and lactate secretion in KMS-11/BTZ cells (Figure 1B,C). Furthermore, the levels of total, surface and secreted ENO1 were also increased in KMS-11/BTZ cells (Figure 1D–H). Our group previously demonstrated, by using an anti-ENO1-specific antibody, that not only intracellular but also extracellular ENO1 (membrane-associated and secreted forms) could contribute to enhanced glycolysis in MM [20]. These findings suggest that metabolic changes, including enhanced glycolysis and elevated ENO1 expression, may contribute to BTZ resistance in the MM cells.

### 3.2. HuL001 Reduces Tumor Growth in BTZ-Resistant MM Xenograft

**Figure 2:**
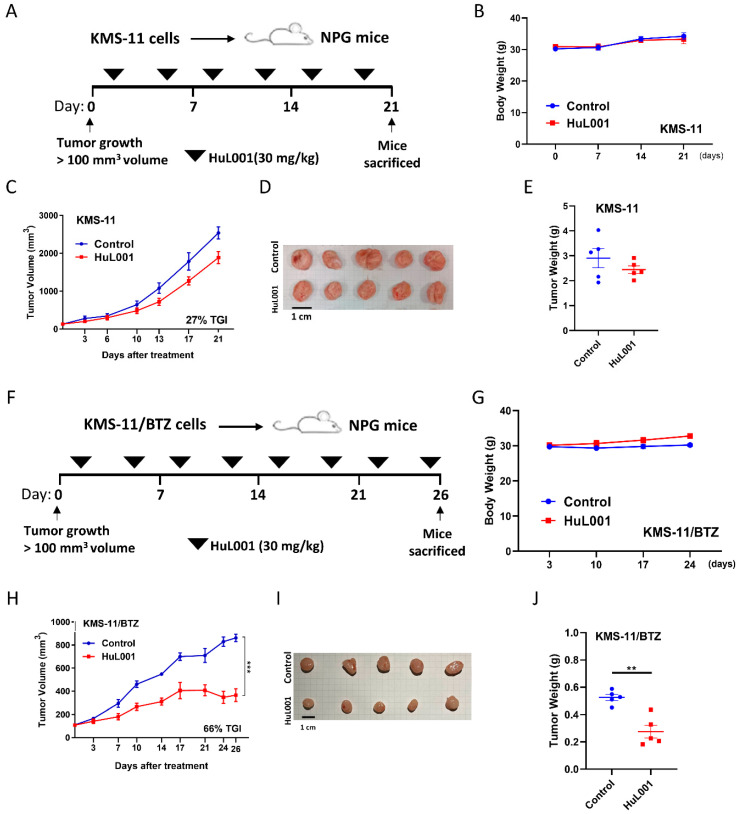
HuL001 reduces tumor growth in a BTZ-resistant MM xenograft. (**A**,**F**) Experimental design: NOD.Cg-Prkdc^scid^ Il2rg^tm1Vst^/Vst mice were subcutaneously implanted with (**A**) KMS-11 or (**F**) KMS-11/BTZ cells on the flank, and treatment started when the tumors reached a mean volume of 100 mm^3^ (set as day 0). Mice were intraperitoneally injected with vehicle control or HuL001 (30 mg/kg, twice weekly). (**B**,**G**) Body weight was measured weekly. (**C**,**H**) The graphs show the average tumor volume for each group over time, and the percentage tumor growth inhibition at the study endpoint was calculated. At the end of study, the tumors were harvested (tumor images are shown in panels (**D**,**I**)), and (**E**,**J**) the tumor weight was measured. Data are presented as the mean ± SEM, pooled from 2 independent in vivo experiments. For each xenograft model, the total group size was *n* = 5 mice per group (vehicle, *n* = 5; HuL001, *n* = 5; total *N* = 10 mice per model; 20 mice overall across the two models). Statistical analysis was performed using two-sided unpaired Student’s *t*-test. ** *p* < 0.01, *** *p* < 0.001.

Our previous study demonstrated that the anti-ENO-specific antibody HuL001 could effectively suppress MM tumor growth in an RPMI-8226 subcutaneous xenograft model [20]. To further explore whether ENO remains a valid target for BTZ-resistant MM, which expresses higher ENO1 levels than BTZ-sensitive MM, HuL001 was administered to mice bearing KMS-11 cell-derived xenograft (CDX) animals (Figure 2A–E) and KMS-11/BTZ (BTZ-resistant) cell-derived animals (Figure 2F–J). HuL001 (30 mg/kg) was administered intraperitoneally in NPG mice twice a week. In the control group, identical experimental procedures were performed, with the exception that PBS (vehicle control) was injected instead of HuL001. Animals were monitored daily, and no significant differences in body weight changes were observed between the groups (Figure 2B,G), indicating minimal treatment-related toxicity. In the KMS-11/BTZ CDX model (Figure 2H–J), in contrast to the KMS-11 CDX model (Figure 2C–E), HuL001 treatment resulted in more significant reductions in tumor volume (effect size = 497 mm^3^, 95% CI = 363–630, TGI = 66%, *p* = 0.0005) and tumor weight compared with the control group at day 26. These findings demonstrate that the ENO1-targeting antibody HuL001 significantly suppresses tumor growth in BTZ-resistant MM xenografts, which exhibit elevated ENO1 expression and enhanced glycolysis compared to their BTZ-sensitive counterparts.

### 3.3. HuL001 Reduces CAF-like Differentiation, Glycolytic Activity and Secretion of IL-6 and VEGF in MM-Educated BMSCs

**Figure 3:**
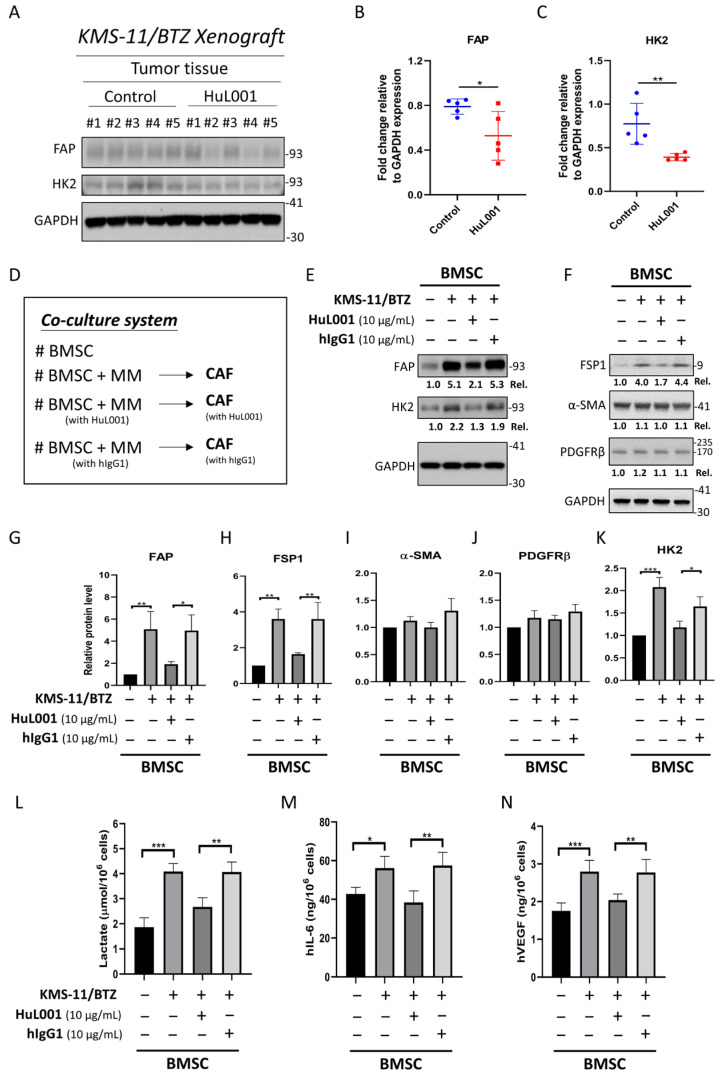
HuL001 reduces CAF-like differentiation, glycolytic activity, and secretion of IL-6 and VEGF in MM-educated BMSCs. (**A**–**C**) NOD.Cg-Prkdc^scid^ Il2rg^tm1Vst^/Vst mice bearing KMS-11/BTZ xenografts were treated with vehicle or HuL001 (30 mg/kg, i.p., twice weekly) starting at ~100 mm^3^. FAP and HK2 were assessed in five representative tumors per group, with GAPDH as a loading control. Panels (**B**,**C**) show the quantified FAP and HK2 levels corresponding to panel (**A**). (**D**) Experimental design: HS-5 BMSCs (adherent culture) were directly co-cultured with KMS-11/BTZ cells (suspension culture) at a 1:2 ratio with or without either HuL001 (10 µg/mL) or human IgG1 (10 µg/mL) for 5 days. Subsequently, suspended KMS-11/BTZ cells were removed, and adherent MM-educated BMSCs (referred as CAFs) were collected for immunoblot analysis or secretion assays. (**E**,**F**) Representative immunoblots from one of three independent biological experiments. Panel (**E**) shows FAP and HK2, and panel (**F**) shows FSP1, PDGFRβ, and α-SMA. (**G**,**K**) Densitometric quantification of FAP and HK2 immunoblot signals corresponding to the experiments represented by panel (**E**), based on three independent biological experiments. (**H**–**J**) Densitometric quantification of FSP1, PDGFRβ, and α-SMA immunoblot signals corresponding to the experiments represented by panel (**F**), based on three independent biological experiments. Quantified protein levels were normalized to GAPDH, and relative expression levels were calculated by comparison with un-educated BMSCs, which were set to 1.0. (**L**–**N**) After removal of suspended KMS-11/BTZ cells, adherent BMSCs were incubated with fresh culture medium for 2 h, and the secretion of (**L**) lactate, (**M**) IL-6, and (**N**) VEGF was measured. The results were normalized to the cell number in each group. Data in panels (**G**–**N**) are presented as the mean ± standard deviation from at least three independent biological experiments. Statistical analyses were performed using two-sided unpaired Student’s *t*-test or one-way analysis of variance with Tukey’s post hoc test, as appropriate. * *p* < 0.05, ** *p* < 0.01, *** *p* < 0.001. Original Western blot images corresponding to the blots shown in this figure are provided in Figure S6.

Since HuL001 suppressed tumor growth in BTZ-resistant xenografts, whether HuL001 exerts direct effects on MM cells was next evaluated. First, HuL001’s impact on MM cell viability in vitro was assessed, and it was found that HuL001 did not alter the viability of either parental KMS-11 (Figure S1A) or BTZ-resistant KMS-11/BTZ cells (Figure S1B). Next, TME components were investigated, particularly CAFs, a critical TME component promoting MM progression. In KMS-11/BTZ xenografts, HuL001 treatment significantly reduced FAP levels, a hallmark marker of CAF differentiation/activation (Figure 3A,B), with quantified FAP/GAPDH values of 0.79 ± 0.07 in the vehicle group and 0.53 ± 0.22 in the HuL001-treated group. Our previous report indicated that decreased glycolysis in MM cells after HuL001 treatment resulted from the reduction in HK2 expression [20]. Therefore, it was reasoned that the increased glycolytic activity observed in KMS-11/BTZ cells (Figure 1C) could be driven by the increase in HK2. The results also revealed that HK2 expression was reduced after HuL001 treatment in KMS-11/BTZ xenografts, supporting the link between HK2-mediated glycolysis and BTZ resistance (Figure 3A,C), with quantified HK2/GAPDH values of 0.77 ± 0.24 in the vehicle group and 0.39 ± 0.04 in the HuL001-treated group. To investigate the impact of HuL001 on BMSCs, a co-culture system of MM cells and BMSCs was established, as illustrated in Figure 3D. The human BMSC line HS-5 with known CAF differentiation potential [29] was directly co-cultured with KMS-11/BTZ cells in the presence of HuL001 or control hIgG1. As shown in Figure 3E–H, co-culture with KMS-11/BTZ cells induced FAP and FSP1 expression in BMSCs, with relative protein levels normalized to GAPDH and expressed relative to un-educated BMSCs (set to 1.0), increasing from 1.0 to 5.09 ± 1.60 for FAP and from 1.0 to 3.61 ± 0.55 for FSP1. Co-treatment with HuL001 markedly attenuated these increases, to 1.92 ± 0.24 and 1.64 ± 0.07, respectively, supporting MM-mediated CAF-like differentiation, as previously reported [30,31]. Unexpectedly, the induction of FAP and FSP1 was markedly attenuated by co-treatment with HuL001 but not by control hIgG1, suggesting an ENO1-specific suppression of the CAF-like population. However, the other CAF markers (α-SMA and PDGFRβ) were not induced in BMSCs co-cultured with KMS-11/BTZ cells (Figure 3F,I,J). This selective marker pattern may reflect CAF heterogeneity and various biological functions, where inflammatory-like fibroblast states can be enriched without a concomitant myofibroblastic (α-SMA-high) program, depending on tumor-derived cues and niche context [32]. CAFs were known to undergo substantial metabolic reprogramming in the TME, often relying on glycolysis as a primary energy source, which is consistent with the Warburg effect [33,34]. In line with these reports, HuL001 also reduced HK2 expression and thus glycolysis in MM-educated BMSCs (Figure 3E,K), with relative HK2 levels normalized to GAPDH and expressed relative to un-educated BMSCs (set to 1.0), decreasing from 2.08 ± 0.22 to 1.18 ± 0.14. This was accompanied by reduced lactate secretion, by decreasing from 4.09 ± 0.32 to 2.68 ± 0.36 μmol/10^6^ cells. Furthermore, HuL001 treatment decreased the secretion of key tumor-promoting factors in MM-educated BMSCs, including lactate (Figure 3L), IL-6 (Figure 3M), and VEGF (Figure 3N), from 4.09 ± 0.32 to 2.68 ± 0.36 μmol/10^6^ cells, 56.20 ± 6.04 to 38.39 ± 5.99 ng/10^6^ cells, and 2.80 ± 0.30 to 2.04 ± 0.16 ng/10^6^ cells, respectively. To assess whether this observation could be replicated in an independent BTZ-resistant MM model, BTZ-resistant RPMI 8226 cells were established, exhibiting higher resistance to BTZ (IC_50_ = 13.9 nM) than parental RPMI 8226 cells (IC_50_ = 3.6 nM) (Figure S2A), followed by co-culture with BMSCs. As shown in Figure S2B–F, co-culture with RPMI 8226/BTZ cells significantly increased FSP1 expression in BMSCs. Relative protein levels, normalized to GAPDH and expressed relative to un-educated BMSCs (set to 1.0), showed that FSP1 increased from 1.0 to 23.97 ± 7.50 (Figure S2B,C), whereas FAP and α-SMA were not appreciably induced (Figure S2B,D,E). Concurrently, PDGFRβ was significantly decreased after co-culture, from 1.0 to 0.57 ± 0.06, and was partially offset by HuL001, to 0.77 ± 0.12 (Figure S2B,F). Collectively, these data indicate an FSP1-biased stromal marker pattern without concordant induction of canonical CAF-associated markers (FAP and α-SMA). Notably, lactate and IL-6 secretion was not appreciably increased in RPMI 8226/BTZ-educated BMSCs (Figure S2G,H), further supporting a restricted, FSP1-biased stromal response rather than broad activation of CAF-associated features. Taken together, across two BTZ-resistant MM models, MM-BMSC co-culture elicited heterogeneous stromal responses with selective CAF-associated marker induction, and HuL001 attenuated these induced changes. Differential CAF-associated marker induction could reflect the level of BTZ-resistance (IC_50_ = 13.9 vs. 43.8 nM), which also led to differential expression of lactate, IL-6 and VEGF in two different BTZ-resistant cell lines.

### 3.4. HIF-1α Contributes to Glycolytic Remodeling and Modulates FAP Expression in MM-Educated BMSCs

**Figure 4:**
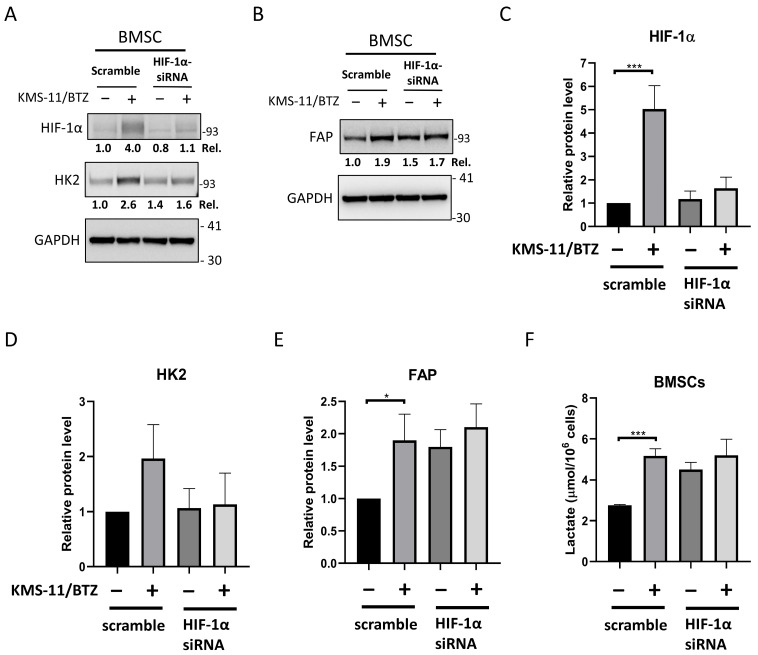
HIF-1α contributes to glycolytic remodeling and modulates FAP expression in MM-educated BMSCs. HS-5 BMSCs were transfected with HIF-1α siRNA or control siRNA (scramble) for 3 days, and then co-cultured with KMS-11/bortezomib cells at a BMSC:MM ratio of 1:2. After removing the suspended MM cells, the adherent BMSCs were analyzed for (**A**) HIF-1α and HK2 at day 1 and (**B**) FAP at day 5, or (**F**) were incubated in fresh medium for 2 h to assess lactate secretion. Panels (**A**,**B**) show representative immunoblots from one of three independent biological experiments, with GAPDH used as a loading control. Panels (**C**,**D**) show densitometric quantification from the same experimental set represented in panel (**A**), based on three independent biological experiments. Panel (**E**) shows densitometric quantification from the same experimental set represented in panel (**B**), based on three independent biological experiments. The signals were normalized to GAPDH, and relative expression levels were calculated by comparison with un-educated BMSCs (MM−, scramble), which were set to 1.0. The results in panels (**C**–**F**) are presented as the mean ± standard deviation. Statistical analyses were performed using one-way analysis of variance with Tukey’s post hoc test. * *p* < 0.05, *** *p* < 0.001. Original Western blot images corresponding to the blots shown in this figure are provided in Figure S6.

Since these CAF-associated changes were accompanied by HK2 induction and glycolytic outputs, whether stromal glycolytic reprogramming is required for CAF-like differentiation was next examined. As shown in Figure 4A,C,D, MM-educated BMSCs exhibited increased HIF-1α protein expression alongside induction of HK2 compared with control BMSCs. Importantly, knockdown of HIF-1α in MM-educated BMSCs substantially reduced HK2 induction, indicating that HK2 upregulation in response to MM education is dependent on HIF-1α signaling. In parallel, MM education significantly increased the expression of FAP, whereas such increase was not observed upon HIF-1α knockdown (Figure 4B,E). Consistent with these findings, extracellular lactate accumulation was elevated in MM-educated BMSCs, but was significantly reduced upon suppression of HIF-1α (Figure 4F). Collectively, these results indicate that HIF-1α-associated glycolytic reprogramming contributes to both HK2 induction and FAP-associated CAF-like differentiation in MM-educated BMSCs.

### 3.5. Extracellular ENO1 Promotes MM-Associated CAF Differentiation of BMSCs via the Plasmin/TGF-β Axis

**Figure 5:**
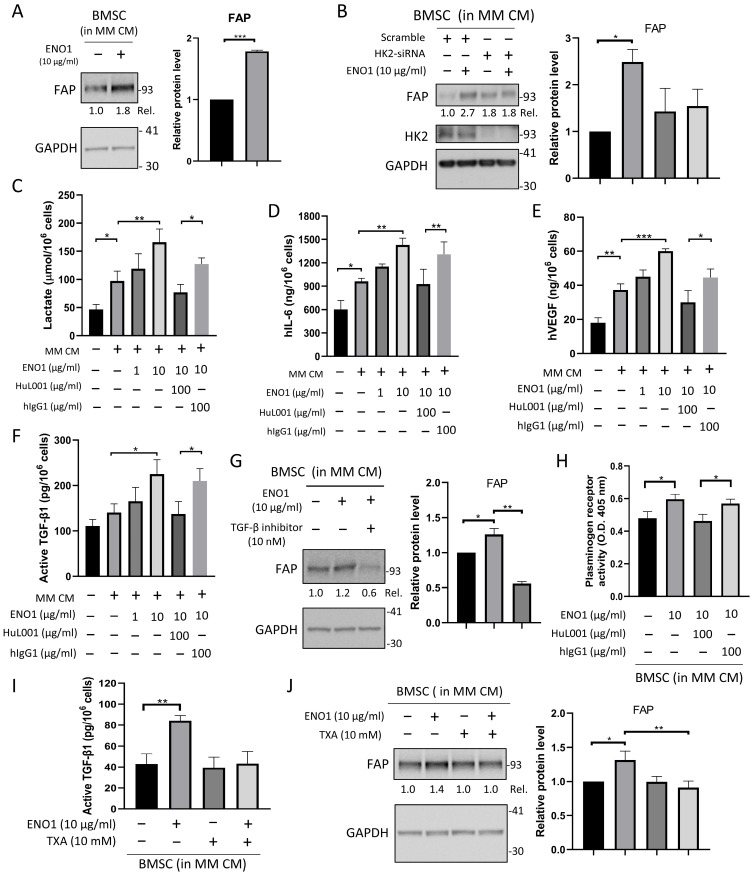
Extracellular ENO1 promotes cancer-associated fibroblast-like differentiation of BMSCs through the plasmin/TGF-β axis. (**A**,**G**,**J**) HS-5 BMSCs were cultured in 50% CM derived from KMS-11/bortezomib cells (MM CM) with or without ENO1 protein, TXA (10 mM) and the TGF-β receptor kinase inhibitor SB431542 (10 nM) for 1 day. (**B**) HS-5 BMSCs were transfected with hexokinase 2 small interfering RNA or scramble control, cultured in 50% MM CM for 3 days, and then treated with ENO1 protein for 1 additional day before immunoblot analysis. In panels (**A**,**B**,**G**,**J**), the left panels show representative immunoblots for FAP, whereas the right panels show densitometric quantification of FAP immunoblot signals from the same experimental sets. FAP signals were normalized to GAPDH, and relative expression levels were calculated by comparison with untreated cells, which were set to 1.0. Quantification in panels (**A**,**B**,**G**) was based on two independent biological experiments, whereas quantification in panel (**J**) was based on three independent biological experiments. Data in the right panels of (**A**,**B**,**G**,**J**) are presented as the mean ± standard deviation. (**C**–**F**,**I**) HS-5 BMSCs were cultured in 50% MM CM with or without ENO1 protein, HuL001 (100 µg/mL), hIgG1 (100 µg/mL), and TXA (10 mM) for 2 days. Supernatants were collected to measure (**C**) lactate, (**D**) interleukin-6, (**E**) vascular endothelial growth factor and (**F**,**I**) active transforming growth factor-β1. The results were normalized to the cell number in each group. (**H**) HS-5 BMSCs were cultured in 50% MM CM with or without ENO1 protein, HuL001 (100 µg/mL), and hIgG1 (100 µg/mL), followed by measurement of plasminogen receptor activity. Data in panels (**C**–**F**,**H**,**I**) are presented as the mean ± standard deviation from three independent biological experiments. Statistical analyses were performed using one-way analysis of variance with Tukey’s post hoc test. * *p* < 0.05, ** *p* < 0.01, *** *p* < 0.001. Original Western blot images corresponding to the blots shown in this figure are provided in Figure S6.

After having established a HIF-1α/HK2-dependent glycolytic requirement for FAP induction, the present study next investigated the upstream extracellular cue(s) in BTZ-resistant MM cells that initiate this stromal programming. The aforementioned results suggested that extracellular ENO1 may modulate CAF differentiation and functions. To further investigate the related mechanism, the proteinase activity associated with extracellular ENO1 and its possible enzymatic target downstream was investigated. To mimic the MM TME in vitro, HS-5 BMSCs were exposed to CM derived from KMS-11/BTZ cells and ENO1 protein. The results indicated that FAP expression was increased in ENO1-treated HS-5 BMSCs compared with untreated cells (Figure 5A), with relative FAP levels normalized to GAPDH and expressed relative to untreated cells (set to 1.0), increasing from 1.0 to 1.79 ± 0.02. Notably, ENO1-induced FAP expression was reduced by HK2-siRNA but not by the scramble control, indicating that glycolysis was probably essential for enhanced FAP expression (Figure 5B). In addition, ENO1 protein treatment dose-dependently induced the secretion of lactate (Figure 5C), IL-6 (Figure 5D) and VEGF (Figure 5E) from HS-5 BMSCs, reaching 166.2 ± 23.58 μmol/10^6^ cells, 1430 ± 85.10 ng/10^6^ cells, and 60.03 ± 1.49 ng/10^6^ cells, respectively, which was reduced by HuL001 co-treatment, to 77.09 ± 13.83 μmol/10^6^ cells, 927 ± 191 ng/10^6^ cells, and 30.00 ± 7.00 ng/10^6^ cells, respectively, confirming the ENO1-specific inhibition. The ENO1-induced secretion of lactate (Figure S3A), IL-6 (Figure S3B) and VEGF (Figure S3C) was similarly reduced by HK2-siRNA treatment.

Previous research implicated TGF-β signaling in CAF activation via upregulating FAP [13]. Consistently, TGF-β markedly upregulated FAP in HS-5 BMSCs (Figure S4A,B). Relative protein levels, normalized to GAPDH and expressed relative to untreated BMSCs (set to 1.0), increased from 1.0 to 5.07 ± 0.68 for FAP and from 1.0 to 3.13 ± 0.32 for PDGFRβ. Conversely, FSP1 and α-SMA levels were reduced (Figure S4A,C,D), from 1.0 to 0.50 ± 0.17 and from 1.0 to 0.77 ± 0.06, respectively, supporting a context-dependent, marker-selective fibroblast activation program. Collectively, these findings raise the possibility that ENO1 may contribute to FAP upregulation, at least partly through modulation of TGF-β signaling, which is known to exist in a latent form requiring activation. In agreement, ENO1 treatment increased active TGF-β levels in HS-5 BMSCs, from 140.3 ± 19.31 to 225.1 ± 31.76 pg/10^6^ cells, which was reduced by HuL001 co-treatment to 137.2 ± 27.37 (Figure 5F). Consistently, FAP expression was significantly inhibited by the TGF-β receptor kinase inhibitor SB431542, which blocked TGF-β signaling in ENO1-treated HS-5 BMSCs (Figure 5G), with relative FAP levels, normalized to GAPDH and expressed relative to the control group (set to 1.0), decreasing from 1.26 ± 0.09 to 0.56 ± 0.03. The ENO1-induced secretion of lactate (Figure S5A), IL-6 (Figure S5B) and VEGF (Figure S5C) was similarly reduced by the TGF-β receptor kinase inhibitor SB431542.

As plasmin is known to activate latent TGF-β [35] by its proteinase activity, the current study next examined whether ENO1-mediated plasmin activation, a complex enzymatic processing from plasminogen to plasmin, contributes to TGF-β activation. As shown in Figure 5H, ENO1 protein enhanced plasminogen receptor activity (plasmin proteinase activity) in HS-5 BMSCs, with OD450 values increasing from 0.48 ± 0.04 to 0.60 ± 0.03, and this increase was inhibited by HuL001 to 0.46 ± 0.04. Notably, TXA treatment reduced both ENO1-induced active TGF-β (Figure 5I) and FAP expression (Figure 5J) in BMSCs cultured in MM CM. Active TGF-β decreased from 84.12 ± 5.03 to 43.26 ± 11.70 pg/10^6^ cells, whereas relative FAP levels, normalized to GAPDH and expressed relative to the control group (set to 1.0), decreased from 1.32 ± 0.13 to 0.91 ± 0.10, consistent with the activity of TXA as a plasmin inhibitor. Collectively, these findings support the hypothesis that the extracellular ENO1-mediated promotion of CAF differentiation and functionality in MM-associated BMSCs is probably triggered by ENO1-mediated proteinase processing of two proteins, namely plasminogen and latent TGF-β, which consequently leads to the generation of active TGF-β after proteolytic cleavage by plasmin.

### 3.6. Promotion Activity of MM Cell Proliferation of MM-Educated BMSCs Is Significantly Reduced After HuL001 Treatment In Vitro and In Vivo

**Figure 6:**
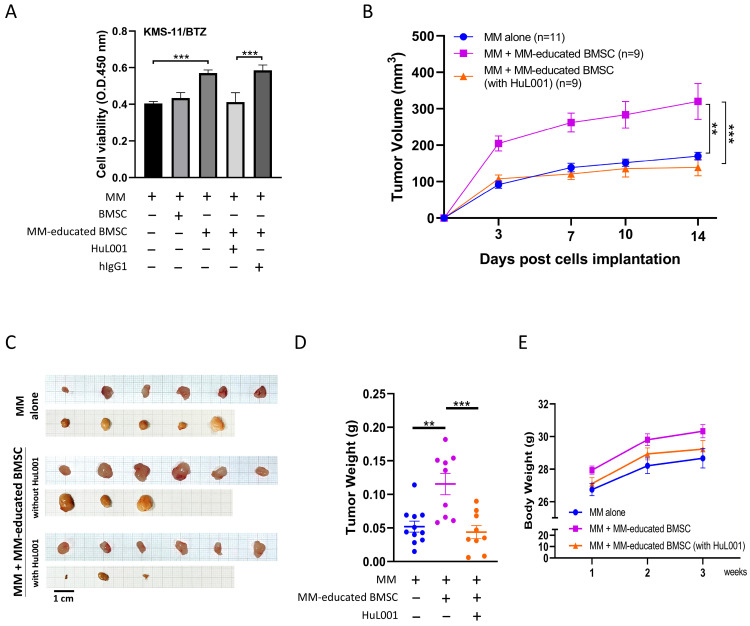
Promotion activity of MM cell proliferation of MM-educated BMSCs is significantly reduced after HuL001 treatment in vitro and in vivo. (**A**) KMS-11/BTZ cells were seeded in the upper chamber of a Transwell insert (0.4-μm pore), while MM-educated BMSCs, pre-treated with either HuL001 (10 μg/mL) or human IgG1 (10 μg/mL), were placed in the lower compartment of the culture wells for 5 days. Next, KMS-11/BTZ cells were collected, and their viability was assessed using the Cell Counting Kit-8 assay. (**B**–**E**) KMS-11/BTZ cells were subcutaneously co-injected with MM-educated BMSCs into NOD.Cg-Prkdc^scid^ Il2rg^tm1Vst^/Vst mice at a 1:2 ratio. Prior to injection, the MM-educated BMSCs were pre-treated with or without HuL001 (10 µg/mL). Each data point is presented as the mean ± SEM, derived from 2 independent biological experiments. (**B**) The graph illustrates the average tumor volumes for each group over time. On day 14, the tumors were harvested and (**C**) photographed, and (**D**) the tumor weight was measured. (**E**) Body weight was measured weekly. The results in panel (**A**) are presented as the mean ± standard deviation from three independent biological experiments. The results in panels (**B**,**D**,**E**) are presented as the mean ± SEM (MM alone, *n* = 11; MM + MM-educated BMSCs, *n* = 9; MM + MM-educated BMSCs + HuL001, *n* = 9; total *N* = 29). Statistical analyses were performed using one-way analysis of variance with Tukey’s post hoc test. ** *p* < 0.01, *** *p* < 0.001.

Based on the aforementioned mechanistic findings, the present study next evaluated whether ENO1 is essential for the promotion of MM cell proliferation by MM-educated BMSCs in the MM co-culturing system and in vivo. MM-educated BMSCs (in the presence of HuL001 or control hIgG1) were indirectly co-cultured with KMS-11/BTZ cells using a Transwell system at a 1:10 ratio. As shown in Figure 6A, MM-educated BMSCs increased the viability of KMS-11/BTZ cells, whereas this effect was significantly reduced when MM-educated BMSCs were pretreated with HuL001.

Implantation of KMS-11/BTZ cells with MM-educated BMSCs in animals led to increased tumor volume and weight, compared with the implantation of MM cells alone (Figure 6B–D). Consistent with the in vitro findings, pretreatment of HuL001 suppressed the tumor promoting effect of MM-educated BMSCs. No significant differences in body weight were observed across groups (Figure 6E). These data suggest that the tumor-supportive function of MM-educated BMSCs is at least partly mediated by extracellular ENO1 in vitro and in vivo.

### 3.7. Combination of HuL001 with LEN Enhances Antitumor Activity in BTZ-Resistant MM Xenografts

**Figure 7:**
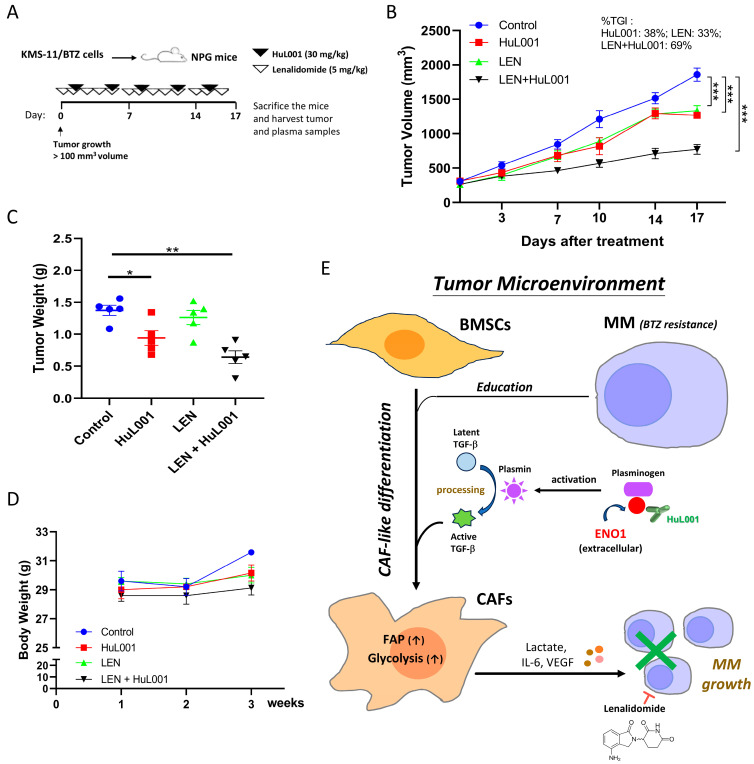
Combination of HuL001 with LEN enhances antitumor activity in BTZ-resistant MM xenografts. (**A**) Experimental design: NOD.Cg-Prkdc^scid^ Il2rg^tm1Vst^/Vst mice bearing KMS-11/BTZ xenografts were treated from day 0 (when tumor volume was ~100 mm^3^) with vehicle, HuL001 (30 mg/kg, i.p., twice weekly), LEN (5 mg/kg, five times/week) or LEN + HuL001. (**B**) The graph shows the average tumor volume for each group over time. The percentage of tumor growth inhibition at the study endpoint was calculated. (**C**) On Day 17, the tumors were harvested, followed by measurement of tumor weight. (**D**) Body weight was recorded weekly. (**E**) Schematic diagram summarizing the effects and possible mechanism of extracellular ENO1 in regulating glycolysis and differentiation of CAFs; production of IL-6, VEGF and lactate; and MM tumor growth. Extracellular ENO1 may promote CAF differentiation, as evidenced by FAP production through the plasmin/TGF-β axis, and enhance tumor-supportive factors, including lactate, IL-6 and VEGF in MM-associated CAF-like cells. Consistently, the aforementioned effects on CAF differentiation and MM tumor growth could be attenuated by the ENO1-specific antibody HuL001. Data are presented as the mean ± standard error of the mean (*n* = 5 mice per group; 4 groups total; total *N* = 20 mice). Statistical analyses were performed using one-way analysis of variance with Tukey’s post hoc test. * *p* < 0.05, ** *p* < 0.01, *** *p* < 0.001.

The current study next evaluated the effect of HuL001 as a combination partner with clinically used agents for BTZ-resistant MM. For patients with MM who develop resistance to BTZ, LEN-based regimens are often adopted as standard of care (SoC) due to their distinct mechanisms of action. Given the common practice of combination therapies in patients with newly diagnosed and RRMM [1], the current study evaluated the therapeutic potential of HuL001 with a unique mechanism of action in combination with LEN. NPG mice bearing KMS-11/BTZ-derived xenografts were treated with HuL001 (30 mg/kg, twice weekly), LEN (5 mg/kg, five times weekly) or their combination (Figure 7A). While HuL001 combined with BTZ showed no therapeutic effect, whereas the combination of HuL001 and LEN significantly reduced tumor volume (effect size = 1086 mm^3^, 95% CI = 794.6 to 1379, TGI = 69%, *p* < 0.0001) and weight. Notably, HuL001 or lenalidomide alone also significantly inhibited tumor growth relative to control, although the magnitude of inhibition was modest compared with the combination (Figure 7B,C). There were no significant differences in body weight across the treatment groups (Figure 7D), indicating minimal treatment-related toxicity. These findings suggest the potential of HuL001 as a promising combination partner to LEN in treating patients with BTZ-resistant MM.

## 4. Discussion

The present study provides evidence for a previously underappreciated role of extracellular ENO1 in promoting CAF differentiation, particularly in the context of BTZ-resistant MM. ENO1 expression was significantly elevated in BTZ-resistant MM cells, associated with enhanced glycolysis and increased secretion of tumor-promoting factors such as lactate, IL-6 and VEGF from CAFs. A novel ENO1-specific mAb, HuL001, effectively reduced tumor growth in a BTZ-resistant MM xenograft model. The inhibitory effect of HuL001 on MM tumor growth appears to involve stromal components, specifically key cellular players within the TME. In line with these observations, HuL001 decreased CAF differentiation and reduced pro-tumorigenic factor secretion in MM-educated BMSCs. To the best of our knowledge, the present study is the first report to demonstrate that extracellular ENO1 promoted CAF differentiation and function via regulating the plasmin/TGF-β axis. Importantly, combining HuL001 with SoC LEN resulted in enhanced antitumor activity in vivo, suggesting that ENO1 inhibition may reprogram the TME to improve therapeutic responsiveness.

Our previous study [20] demonstrated that extracellular ENO1 promoted MM tumor growth possibly via positively regulating glycolysis and its associated pro-cancer activities in MM cells. HuL001 modestly inhibited MM cell proliferation in vitro (in RPMI 8226 and U266 cells), which suggests that direct proliferation inhibition alone is unlikely to account for the magnitude of tumor suppression observed in vivo. Of note, no inhibitory effect on MM cell proliferation was observed in KMS-11 or KMS-11/BTZ cells (Figure S1). It is reasonable to hypothesize that the anticancer effects of HuL001 do not only affect cancer cells but also their interactive TME players such as CAFs. Therefore, an MM-BMSC co-culturing system was introduced to study the interaction between MM cells and CAFs and the resulting tumor growth. Metabolic alterations within the TME drive MM progression and therapeutic resistance [27,36,37]. Importantly, BTZ resistance can also result from tumor-intrinsic alterations in the proteasome machinery, including mutations and/or upregulation of the proteasome β5 subunit (PSMB5), which have been associated with reduced BTZ binding and acquired resistance [38]. PSMB5 variants have also been reported in samples of patients with relapsed/refractory MM, with functional validation supporting their contribution to proteasome inhibitor resistance [39]. Accordingly, an important future direction is to determine whether ENO1-associated stromal activation and HuL001 responsiveness correlate with established genetic resistance determinants (PSMB5 status), and to investigate a potential crosstalk with proteasome activity and broader proteostasis stress responses [40]. Given the established roles of CAFs in MM progression and therapy resistance, the present data position extracellular ENO1 as an upstream driver of CAF-like metabolic remodeling and pro-tumor secretory programs in MM-educated BMSCs (Figure 5 and Figure S3). The current results provide new mechanistic insights into ENO1-driven metabolic crosstalk between MM cells and CAFs. It seems reasonable to target ENO1 for disruption of a metabolic feedback loop that sustains CAF-mediated tumor support. Consistently, Ray et al. [19] identified ENO1 as an immunometabolic target, demonstrating that pharmacological inhibition of ENO1 alleviated immune suppression by disrupting the metabolic activity of pDCs. The crosstalk and metabolite transfer between MM cells and CAFs remain incompletely understood. One hypothetical framework is the complex ‘reverse Warburg effect’, in which glycolytic SCs provide metabolites that support adjacent cancer cells, thereby promoting ATP production, proliferation and survival [41].

Extracellular ENO1 is increasingly recognized as a prototypical ‘moonlighting’ protein that contributes to cancer biology beyond its canonical cytosolic glycolytic function [42]. In multiple solid tumors, ENO1 can relocalize to the cell surface, where it acts as a plasminogen receptor, facilitating pericellular plasmin generation, extracellular matrix remodeling, and downstream programs that support invasion, angiogenesis and metastasis [42,43]. ENO1 has also been reported to traffic to other cellular compartments, including the nucleus as c-Myc promoter-binding protein 1, highlighting context-dependent non-glycolytic activities that may shape tumor behavior and host responses [42,43]. The present study extended the aforementioned ‘moonlighting’ concept to MM by linking extracellular ENO1 to stromal activation and CAF-like differentiation within the BM niche. Exosome-derived ENO1 has been shown to regulate integrin α6β4 expression, promoting tumor growth and metastasis in HCC [21], while BMSC-derived exosomes facilitate MM progression [44]. Importantly, accumulating evidence suggests that extracellular or cell-surface ENO1 is not restricted to MM, as it has been implicated in tumor progression and TME interactions in multiple solid tumors (including glioblastoma, lung adenocarcinoma, sarcoma/osteosarcoma, HCC, as well as melanoma, breast, colorectal, pancreatic, bladder, esophageal, prostate and cervical cancer) and hematological malignancies (such as MM and acute myeloid leukemia) [21,45,46]. However, the downstream consequences of extracellular ENO1 appear to be context dependent, and shaped by tumor type and microenvironmental composition. These findings suggest the possibility that extracellular ENO1 in the MM microenvironment may mediate crosstalk between cancer cells and stromal fibroblasts, triggering their differentiation into CAFs. However, although Figure 4 supports a role for HIF-1α in HK2 induction and FAP-associated stromal changes in MM-educated BMSCs, the direct mechanistic association between extracellular ENO1 signaling and the HIF-1α/HK2 glycolytic program remains to be clarified. The TGF-β signaling pathway, a well-documented driver of CAF differentiation [47], supports paracrine crosstalk between CAFs and MM cells leading to MM progression [8]. TGF-β is generally considered to be an important programmer of immunosuppressive TMEs that promote MM cell survival, invasion and metastasis [7]. Given the finding of ENO1-driven CAF differentiation, it is conceivable that ENO1 may be involved in the TGF-β pathway to regulate the TME. The activation of TGF-β is tightly regulated by various signals, including proteolytic cleavage by proteases [48] such as plasmin [35]. However, the efforts to pursue drugs targeting TGF-β have been disappointing thus far due to its complex biology and unwanted toxicity. Notably, ENO1 functions as a plasminogen receptor leading to plasmin activation [16]. ENO1-induced FAP expression is reduced by a TGF-β inhibitor (Figure 5G), suggesting that ENO-mediated regulation of CAF differentiation could be upstream of TGF-β signaling. In addition, plasminogen receptor activity (plasmin activity) and, concomitantly, the level of TGF-β are downregulated by HuL001 (Figure 5H,I). In agreement with these observations, the levels of TGF-β and FAP were also reduced by TXA treatment, a plasminogen receptor inhibitor (Figure 5I,J). Together, these findings support an ENO1/plasmin/TGF-β axis as a plausible mechanism linking extracellular ENO1 to CAF-associated remodeling in MM (Figure 7E).

Consistent with prior reports linking ENO1 to glycolytic reprogramming and stress adaptation [43,45], the current data suggest that ENO1 is associated with metabolic features of BTZ-resistant MM (Figure 1B,C). These observations are consistent with prior studies linking ENO1 expression to chemoresistance in solid tumors [49,50]. For example, in lung and breast cancer, ENO1-associated glycolysis has been implicated in supporting the survival of drug-resistant cell populations. However, the current results only suggest a link between ENO1-enhanced glycolysis with BTZ resistance, but no causal association was established in vitro. It also remains unknown whether the TME, involving CAFs, could contribute to the BTZ resistance of MM cells in vivo.

From a translational perspective, current MM management primarily relies on tumor-directed agents (proteasome inhibitors, immunomodulatory drugs and anti-CD38 antibodies), with BCMA-directed cellular and bispecific therapies increasingly being used in relapsed/refractory disease [51]. In this context, HuL001 offers a mechanistically distinct, microenvironment-directed strategy by targeting extracellular ENO1, which is consistent with an upstream role of the ENO1/plasmin/TGF-β axis in CAF-associated remodeling. Since direct TGF-β inhibition has been challenging clinically, targeting an upstream activator such as ENO1 may provide a novel approach [52]. Future studies should define biomarkers (surface/extracellular ENO1), establish pharmacodynamics and safety, and test rational combinations in patient-derived and immunocompetent models. While ENO1 targeting shows promise in treating MM [19,20], several limitations exist in the current study. First, CAF-like stromal responses were primarily modeled using the HS-5 BMSC line and were not validated in patient-derived primary CAFs from MM BM; thus, confirmation in patient-derived samples will be important to capture inter-patient heterogeneity and to strengthen translational feasibility. Second, current experimental models, including BTZ-resistant MM xenografts and MM-educated BMSCs in vitro assays, may not fully reproduce the human TME, and these results should be further verified using patient-derived and/or humanized models. Third, the current study also lacks assessment of and insight into the therapeutic effects of the ENO1 mAb on immune dynamics [37], which is crucial for clinical translation. Because HuL001 is a humanized IgG1 antibody, the current study did not include an immune-competent model suitable for fully evaluating its immune-mediated effects in vivo. Future studies in adequately powered syngeneic and/or humanized models are warranted to determine whether HuL001 influences antitumor immune responses within the TME. Fourth, a future direction is to directly test the ENO1/plasmin/TGF-β axis in vivo by inhibiting plasmin activity (genetically or pharmacologically) to establish causality under physiological conditions. In addition, direct pharmacodynamic evaluation of tumor plasmin activity and active TGF-β in vivo will be important to further strengthen target-engagement assessment. Finally, combination treatment studies remain limited, warranting exploration of regimens with proteasome inhibitors, CD38-targeting therapies, CAR-T or ADCs. In addition, toxicity assessment in the current study was limited and was primarily based on body weight monitoring, without more comprehensive safety evaluation. Furthermore, reversibility of the observed effects after HuL001 withdrawal was not examined in the current study. Addressing these gaps with various animal models, further mechanistic studies and expanded combination strategies are essential to fully harness the clinical potential of ENO1-targeting therapeutic strategy in MM, as observed in other cancer types [46].

## 5. Conclusions

The present study identified extracellular ENO1 as an important contributor to TME metabolic remodeling and stromal dynamics in MM, making it a potential therapeutic target for CAFs and BTZ resistance. Cancer-associated neutrophils, fibroblasts and macrophages are responsible for the complexity of the TME, with their secreted pro-cancer molecules, making TME immunosuppressive and prone to developing drug resistance. It is attractive but difficult to develop a TME-targeting therapeutic agent due to the complex stromal milieu, involving various cell types and their interactive effector molecules. The present findings suggest that ENO1 may be a key upstream regulator of TGF-β in the TME, a pathway that remains challenging to inhibit pharmacologically. However, additional studies are required to validate the aforementioned hypothesis in MM and other cancer types (Figure 7E).

## Data Availability

The data supporting the findings of this study have been deposited in Zenodo at https://zenodo.org/records/19222583?preview=1&token=eyJhbGciOiJIUzUxMiJ9.eyJpZCI6IjIxNzEyMDBiLThmNjUtNDA3ZS1hZjlhLWY3Y2ZkMjU2ZTIyNyIsImRhdGEiOnt9LCJyYW5kb20iOiJiZmRhNDBiM2Q3ODg2NDMzZmM1MDBjZGE3NzU4Zjg5YSJ9.Hdpom4DbW3q0y3AeJHi6M6uVHyBCxebj8y1Bjoe9jJCv8S0PxERgN3yBPmvfpWNPbPoBQglATmEtIKsB6y3yKQ. (accessed on 29 April 2026). The deposited dataset includes the source data underlying main and Supplementary figures presented in this study.

## Supplementary Materials

The following supporting information can be downloaded at: https://www.mdpi.com/article/10.3390/cancers18091467/s1, Figure S1: HuL001 may not affect cell viability in multiple myeloma cells; Figure S2: RPMI 8226/BTZ induces an FSP1-biased stromal marker pattern in BMSCs, which is modulated by HuL001; Figure S3: Extracellular ENO1 promotes glycolytic activity and secretion of IL-6 and VEGF via HK2 in MM-associated CAF-like differentiation of BMSCs; Figure S4: TGF-β differentially regulates CAF-associated markers in HS-5 BMSCs; Figure S5: Extracellular ENO1 promotes glycolytic activity, and secretion of IL-6 and VEGF via TGF-β signaling in MM-associated CAF-like differentiation of BMSCs; Figure S6: Original Western blot images.
