# Supplementary material for: Extracellular Enolase-1 Promotes CAF-Associated Stromal Reprogramming via the Plasmin/TGF-β Axis in Multiple Myeloma
Source: Cancers (Basel). 2026 May 2;18(9):1467. doi: 10.3390/cancers18091467 (PMC13163094; doi:10.3390/cancers18091467)
Supplement: Supplementary file 1 [file cancers-18-01467-s001.zip › cancers-4254102-supplementary.pdf]

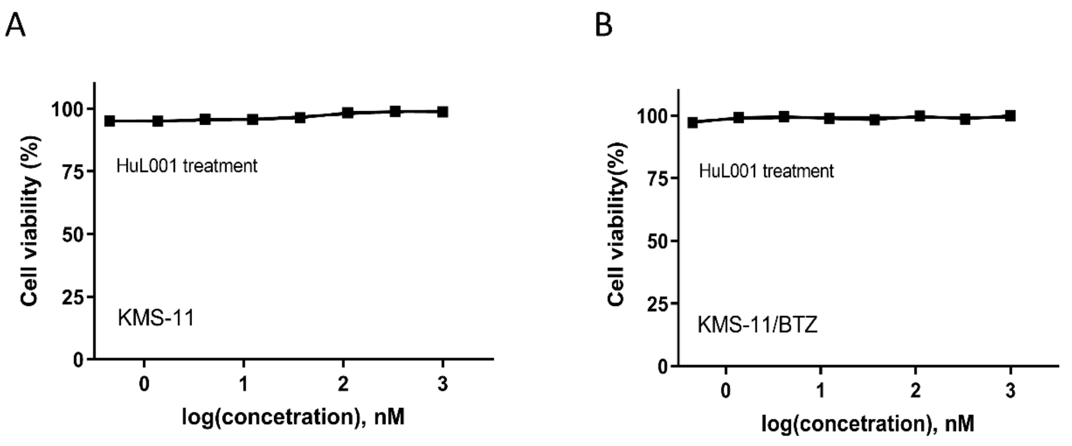

Figure S1. HuL001 may not affect cell viability in multiple myeloma cells. (**A**) KMS-11 and (**B**) KMS-11/bortezomib cells were incubated with various doses of HuL001 for 3 days. The inhibition of cell proliferation was measured by Cell Counting Kit-8 assay.

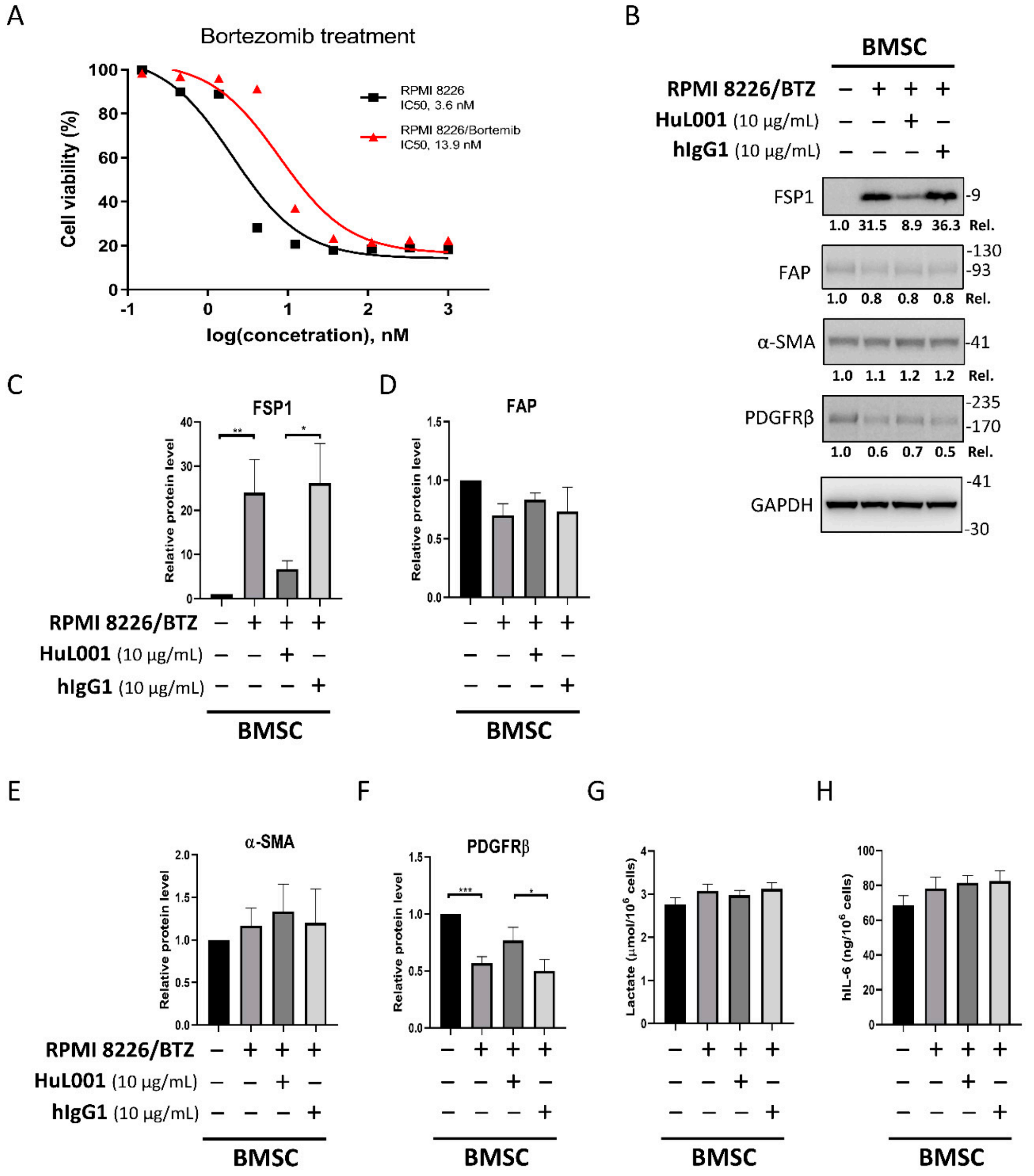

Figure S2. RPMI 8226/BTZ induces an FSP1-biased stromal marker pattern in BMSCs, which is modulated by HuL001. (**A**) RPMI 8226 and RPMI 8226/BTZ cells were incubated with increasing concentrations of BTZ for 3 days, and cell proliferation inhibition was then assessed by Cell Counting Kit-8 assay. **(B**) HS-5 BMSCs were co-cultured with RPMI 8226/BTZ cells at a 1:2 ratio ± HuL001 (10 μg/ml) or human IgG1 (10 μg/ml) for 5 days. After removing the suspended MM cells, adherent BMSCs were immunoblotted for FAP, FSP1, PDGFRβ and α-SMA (GAPDH control) detection, or incubated in fresh medium for 2 h to measure (**G**) lactate

and (**H**) interleukin-6 secretion. Panel **B** shows representative immunoblot results from one of three independent biological experiments. Panels **C-F** show the densitometric quantification of (**C**) FAP, (**D**) FSP1, (**E**) PDGFRβ and (**F**) α-SMA in BMSCs from the same experimental set, based on three independent biological experiments. The signals were normalized to GAPDH, and the relative expression levels were calculated by comparison with un-educated BMSCs, which were set to 1.0. Data in panels C-H are presented as the mean ± standard deviation from three independent biological experiments. Statistical analyses were performed using one-way analysis of variance with Tukey's post hoc test. *$P<0.05$, **$P<0.01$, ***$P<0.001$. Original Western blot images corresponding to the blots shown in this figure are provided in Fig. S6.

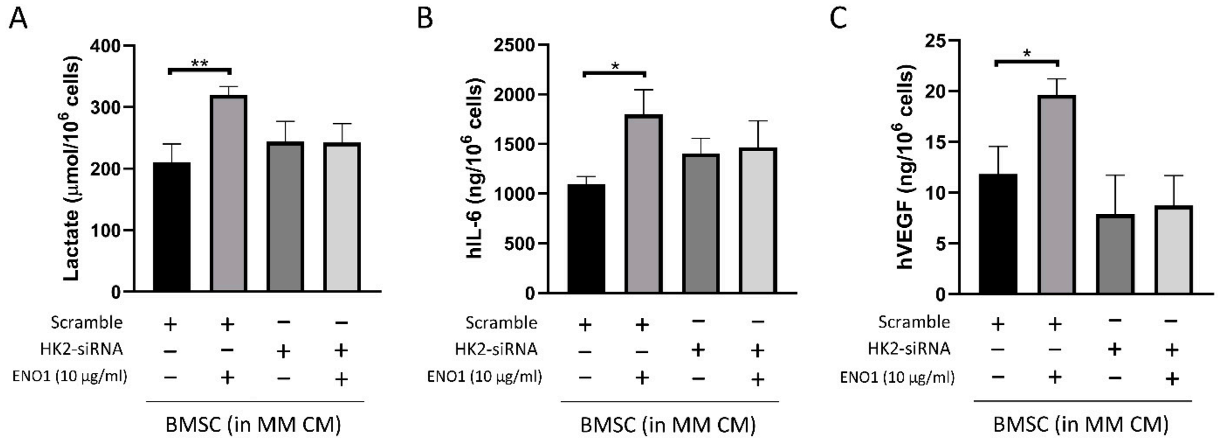

Figure S3. Extracellular ENO1 promotes glycolytic activity and secretion of IL-6 and VEGF via HK2 in MM-associated cancer-associated fibroblast-like differentiation of BMSCs. HS-5 BMSCs were transfected with HK2 small interfering RNA or scramble control, cultured in 50% MM conditioned medium for 3 days, and then treated with recombinant ENO1 for an additional 2 days. The levels of (**A**) lactate, (**B**) IL-6 and (**C**) VEGF in the culture medium were measured and normalized to the cell number. The results were expressed relative to untreated cells (ENO1-, scramble; set to 1.0) and are presented as the mean ± standard deviation from three independent biological experiments. Statistical significance was assessed using a two-sided unpaired Student's t-test. $^{*}P<0.05$, $^{**}P<0.01$.

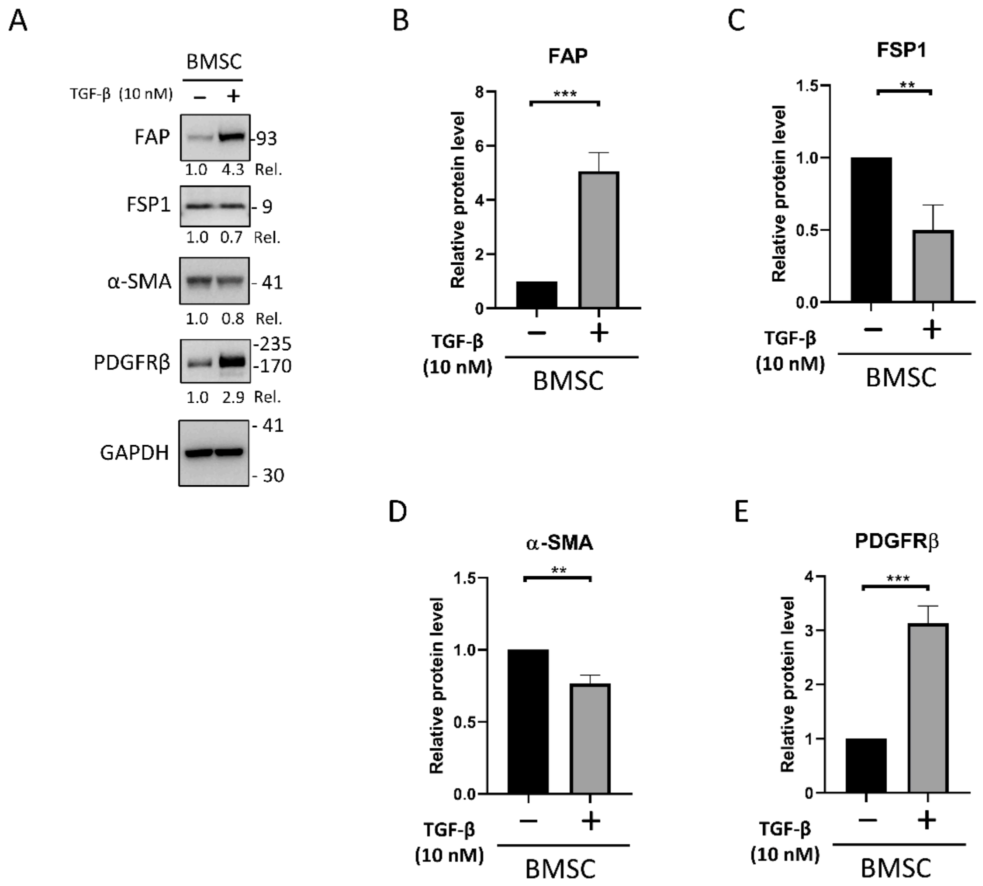

Figure S4. TGF-β differentially regulates cancer-associated fibroblast-associated markers in HS-5 BMSCs. HS-5 BMSCs were treated with TGF-β (10 nM) for 5 days and then subjected to immunoblotting for FAP, FSP1, PDGFRβ and α-SMA. (**A**) Representative immunoblots from one of three independent biological experiments, with GAPDH used as a loading control. Panels **B**-**E** show densitometric quantification of the indicated proteins from the same experimental set, based on three independent biological experiments. The signals were normalized to GAPDH, and relative expression levels were calculated by comparison with untreated control BMSCs, which were set to 1.0. Data are presented as the mean ± standard deviation. Statistical analysis was performed using a two-sided unpaired Student's t-test. **P<0.01, ***P<0.001. Original Western blot images corresponding to the blots shown in this figure are provided in Fig. S6.

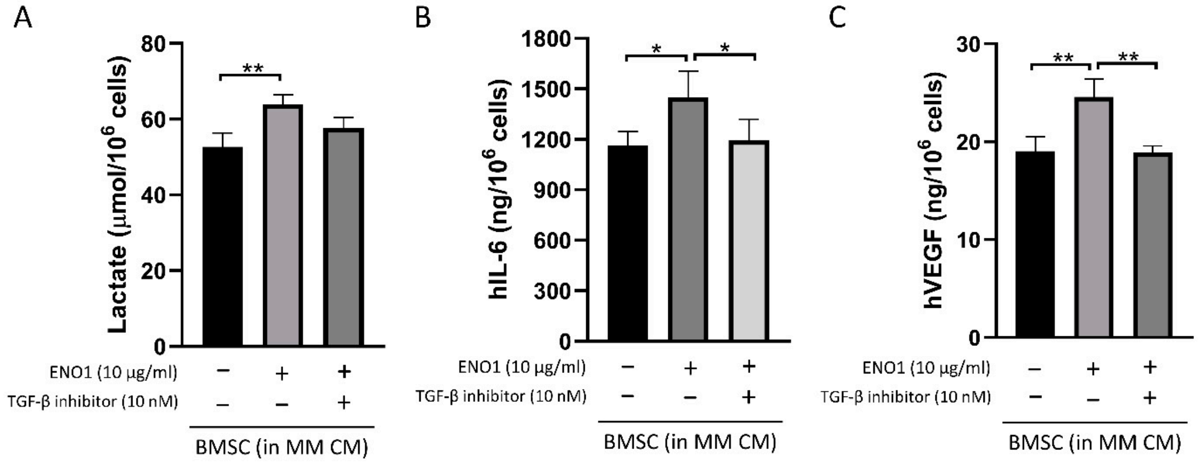

Figure S5. Extracellular ENO1 promotes glycolytic activity, and secretion of IL-6 and VEGF via transforming growth factor-β signaling in MM-associated cancer-associated fibroblast-like differentiation of BMSCs. HS-5 BMSCs were cultured in 50% MM conditioned medium with or without ENO1 proteins and SB431542 (10 nM) for 2 days. The levels of (**A**) lactate, (**B**) IL-6 and (**C**) VEGF in the culture medium were measured. The results were normalized to the cell number in each group. Data are presented as the mean ± standard deviation of three independent biological experiments. Statistical analysis was performed using one-way analysis of variance with Tukey's post hoc test. $^{*}P<0.05$, $^{**}P<0.01$.

Fig. 1E

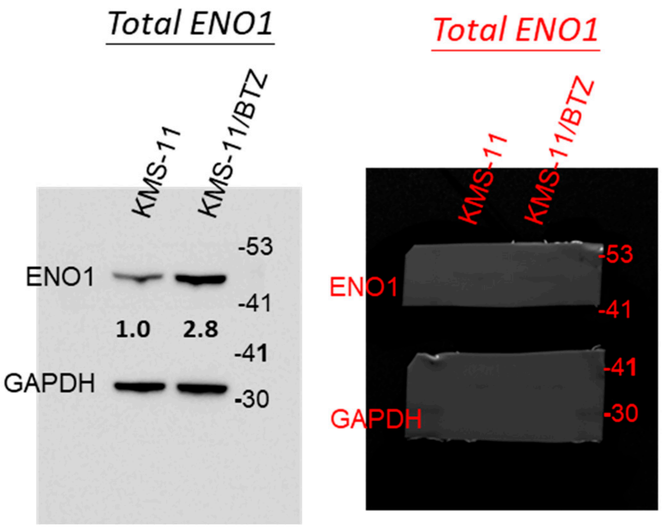

Figure S6. Original Western blot images and preserved membrane sections.

The most complete original images available for the blots shown in Figs. **1E, 3A, 3E-F, 4A-B, 5A-B, 5G, 5J, S2B**, and **S4A** are provided. Because the membranes were cut according to the expected molecular weight range of each target protein prior to primary antibody incubation, full-length images of the entire original membranes were not preserved for all experiments. The preserved membrane sections corresponding to the probed molecular weight ranges are shown, and the available molecular weight marker positions are indicated.

Fig. 3A

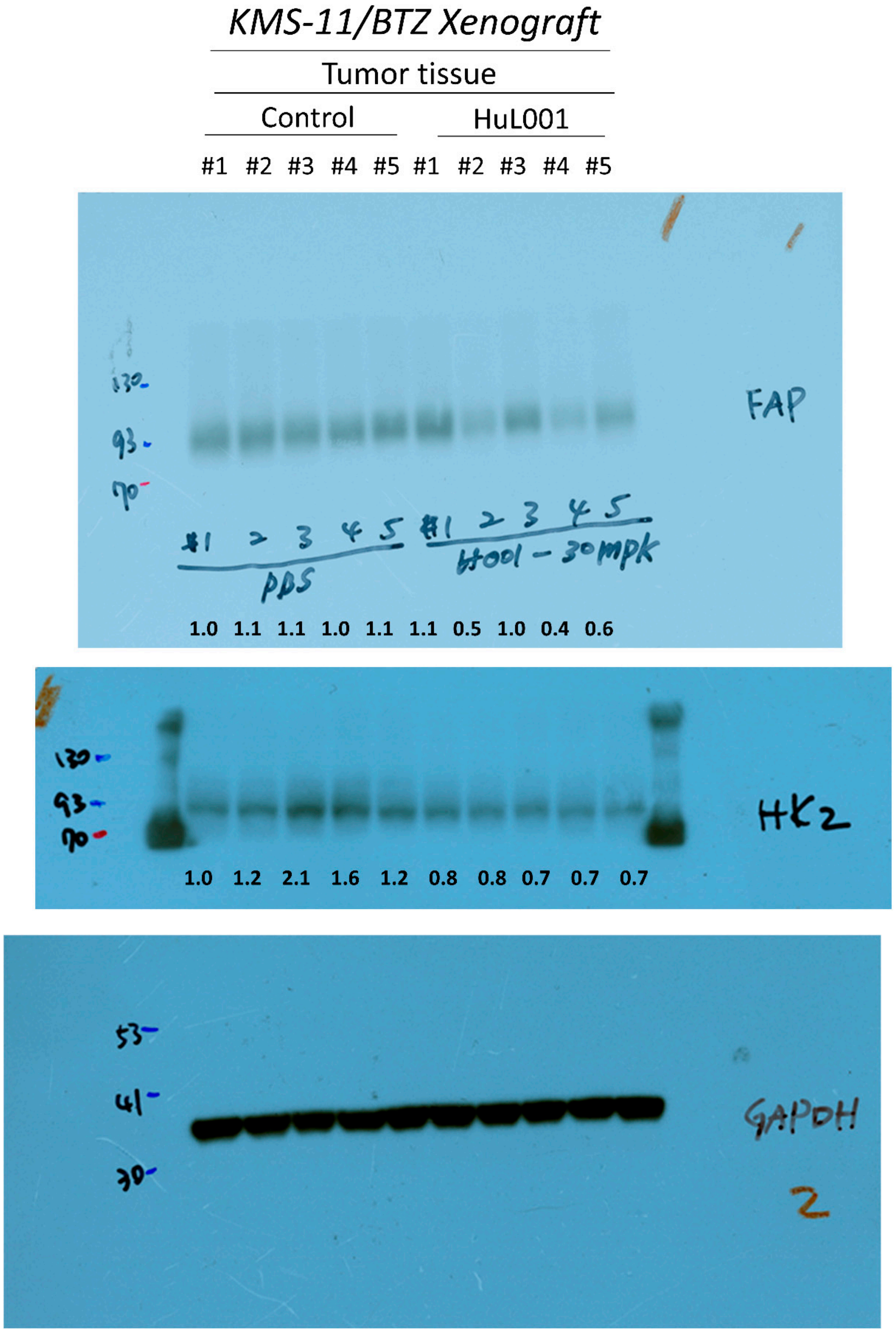

Figure S6 (continued 1).

Fig. 3E

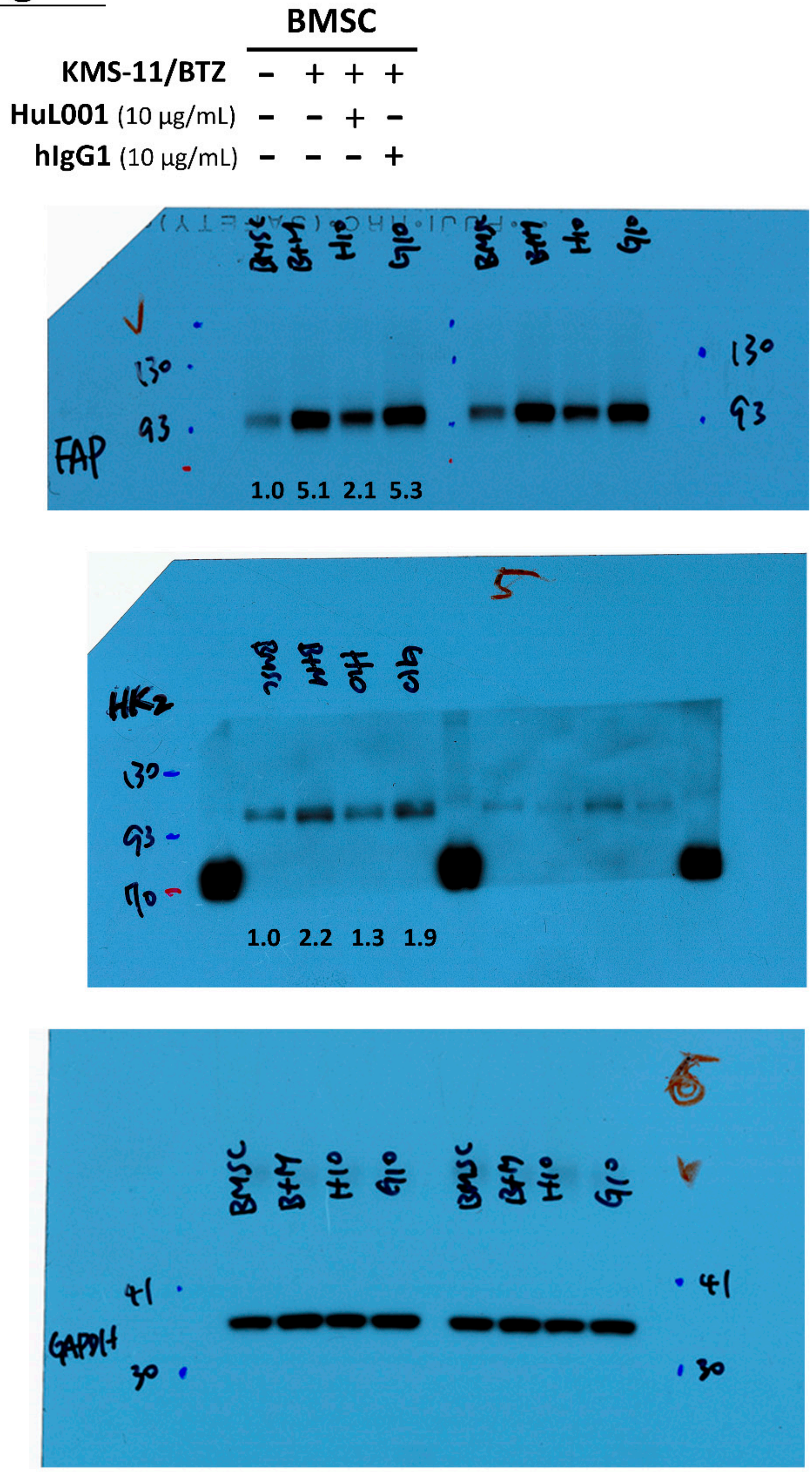

Figure S6 (continued 2).

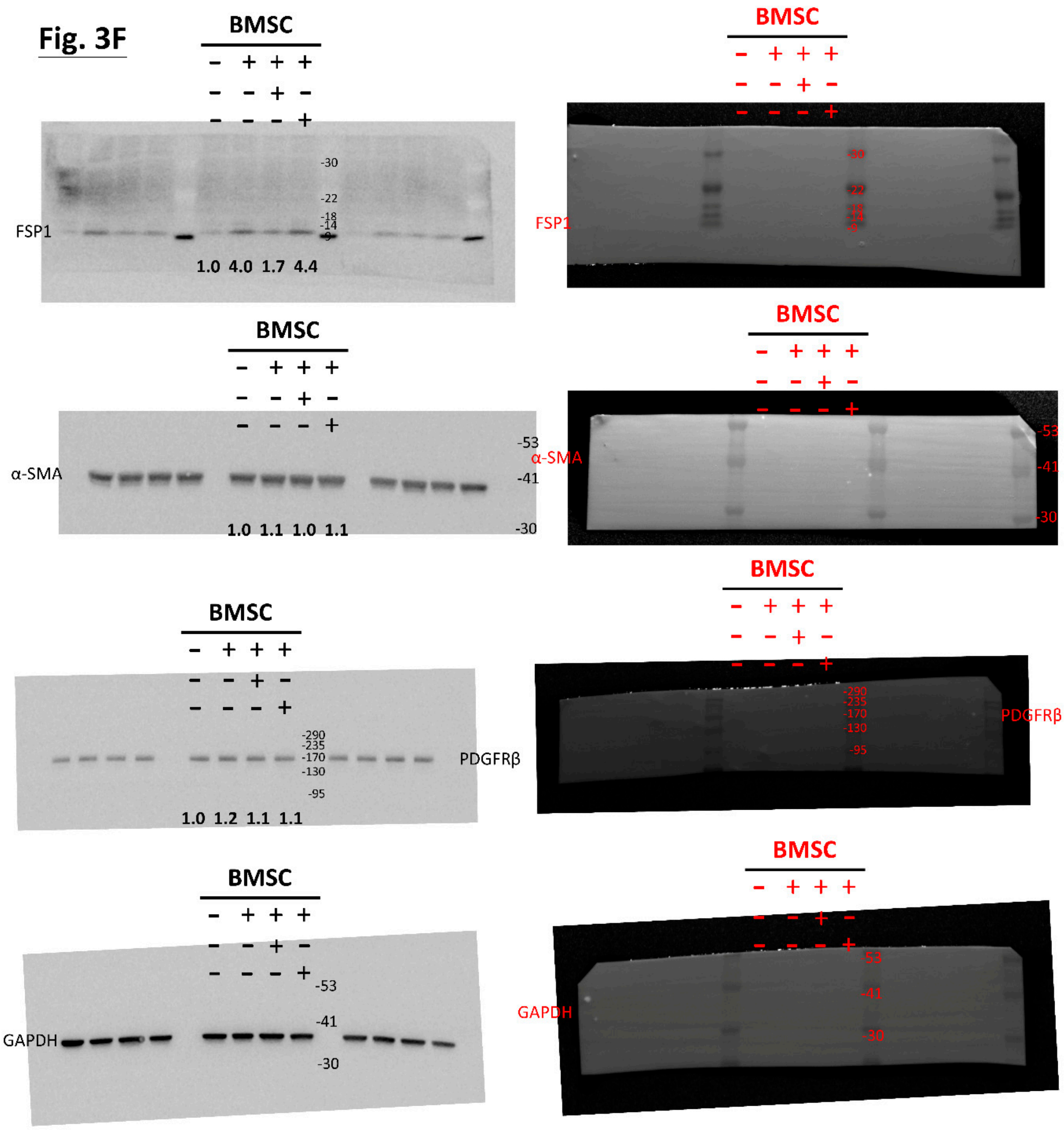

Figure S6 (continued 3).

Fig. 4A

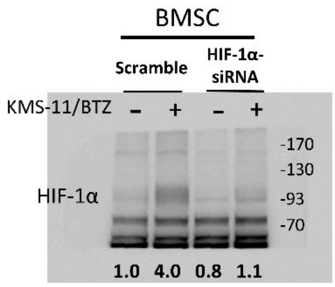

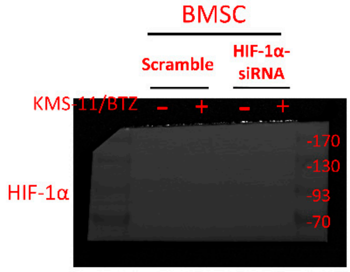

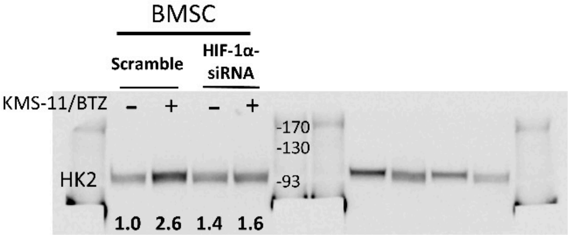

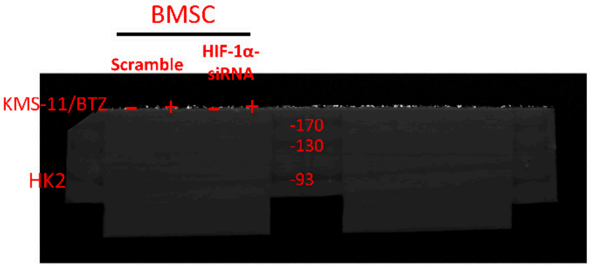

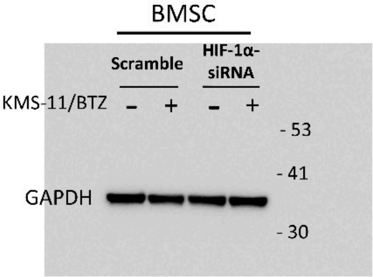

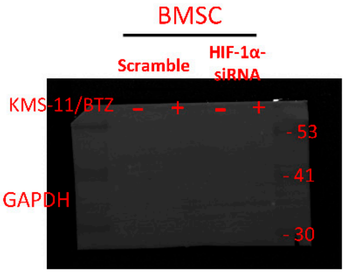

Figure S6 (continued 4).

Fig. 4B

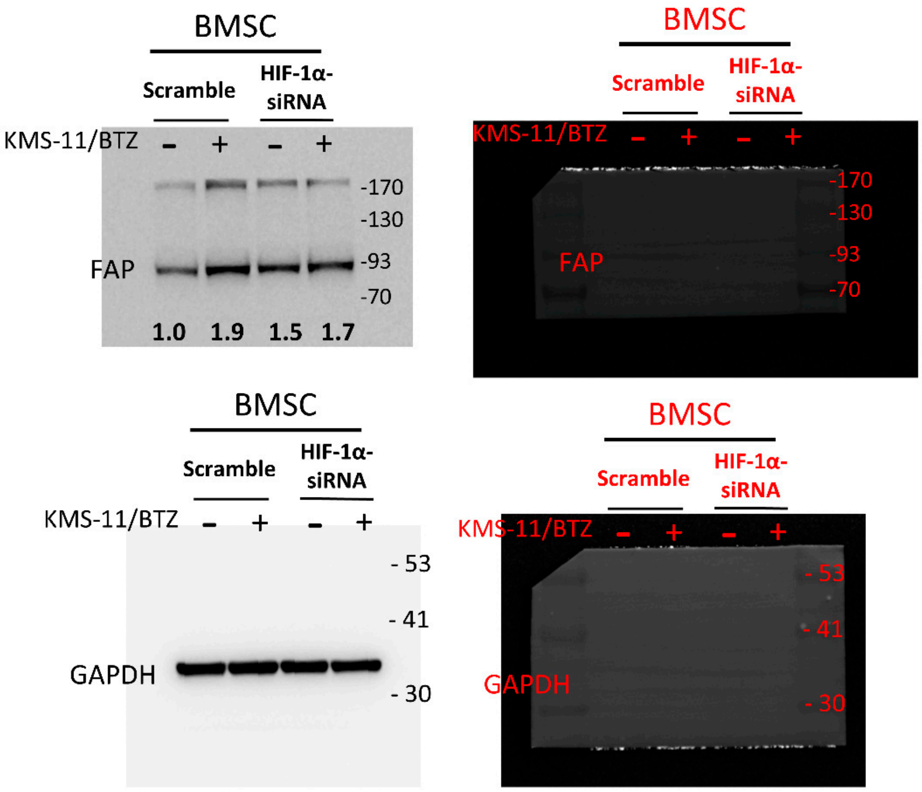

Figure S6 (continued 5).

Fig. 5A

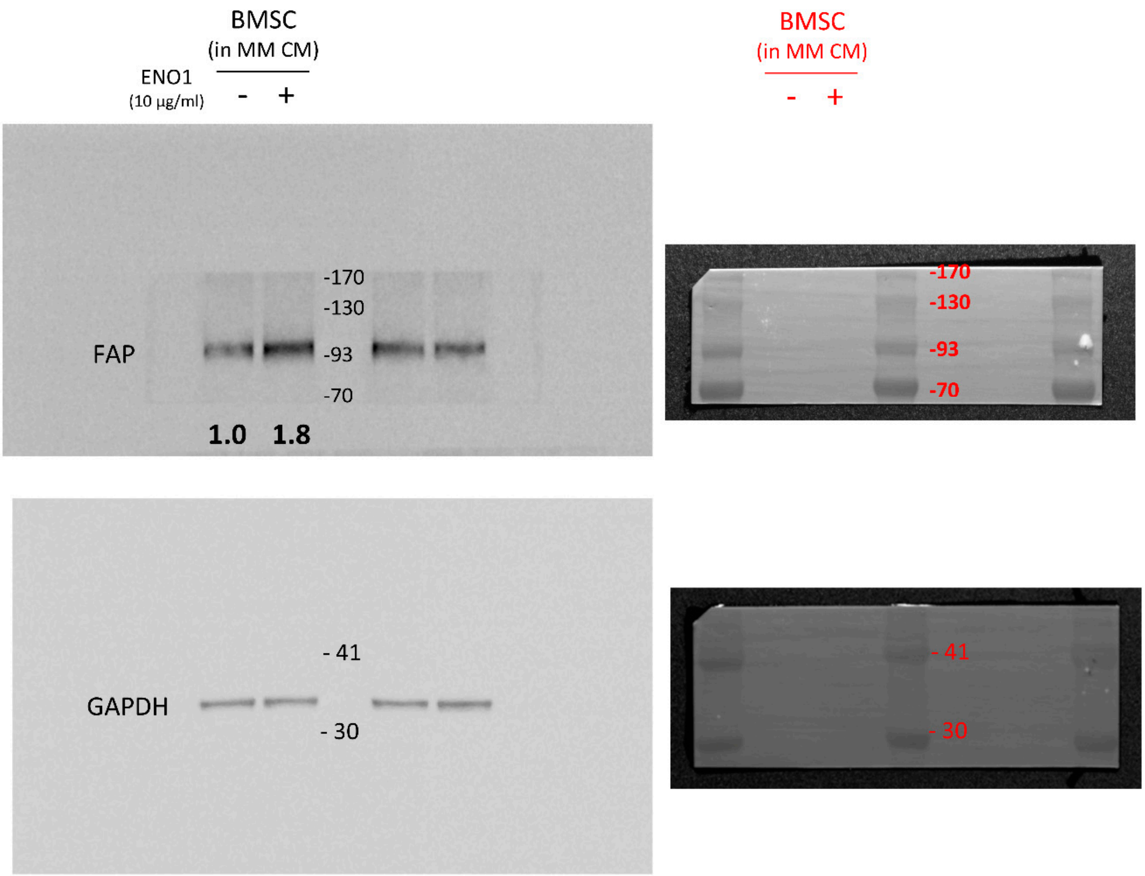

Figure S6 (continued 6).

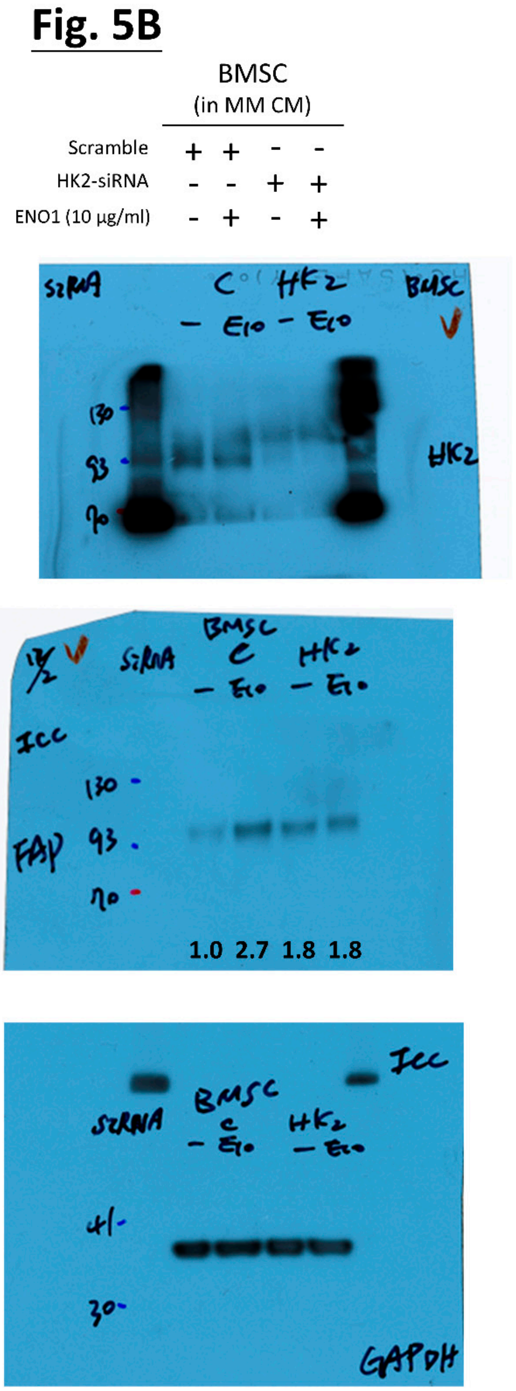

Figure S6 (continued 7).

Fig. 5G

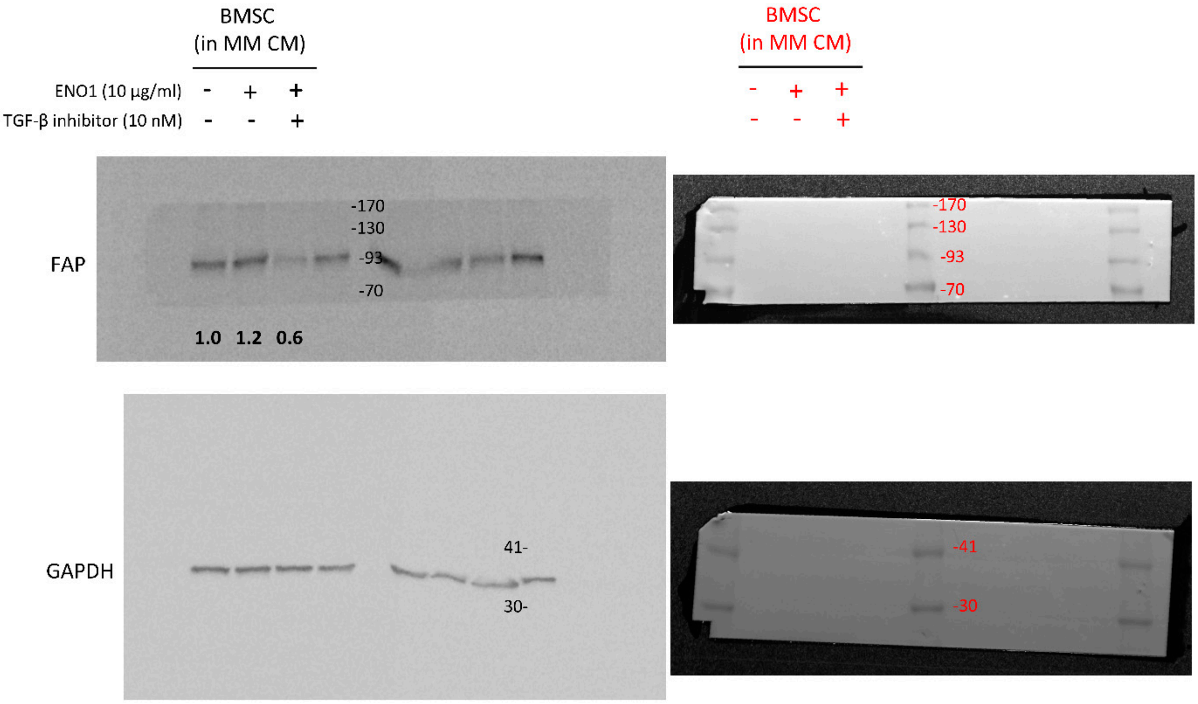

Figure S6 (continued 8).

Fig. 5J

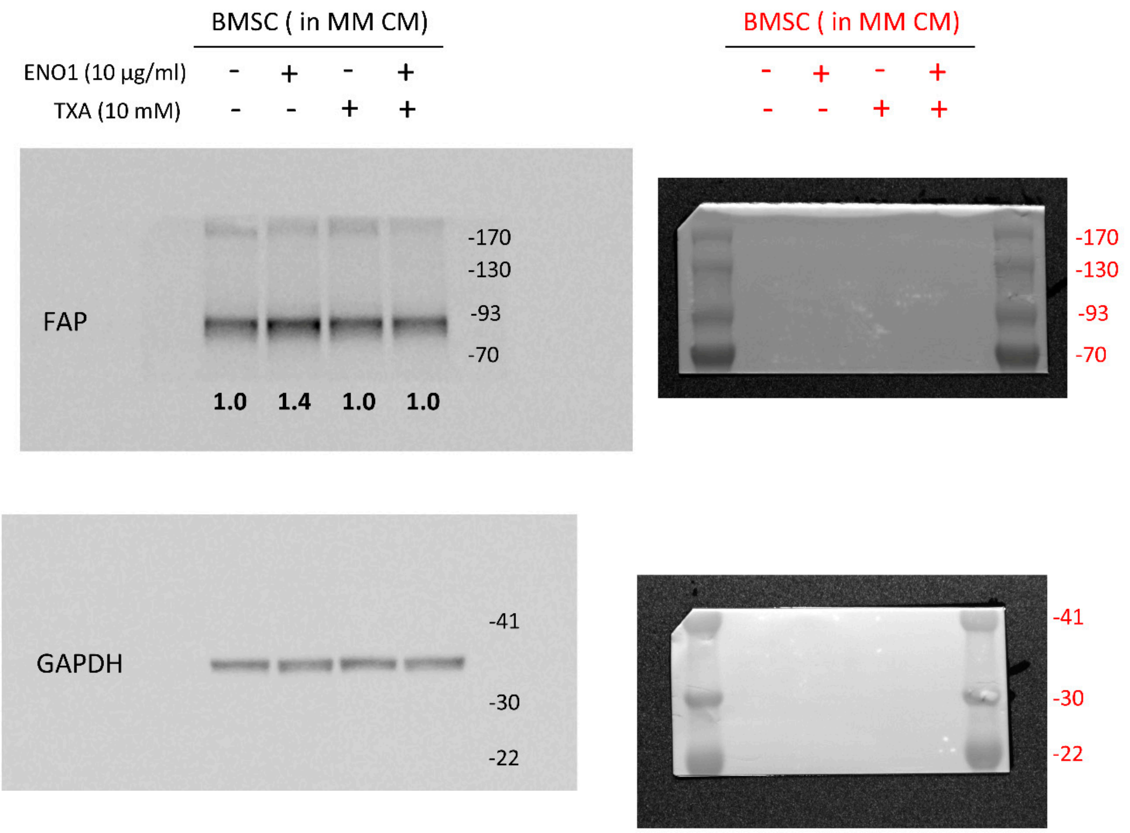

Figure S6 (continued 9).

Fig. S2B

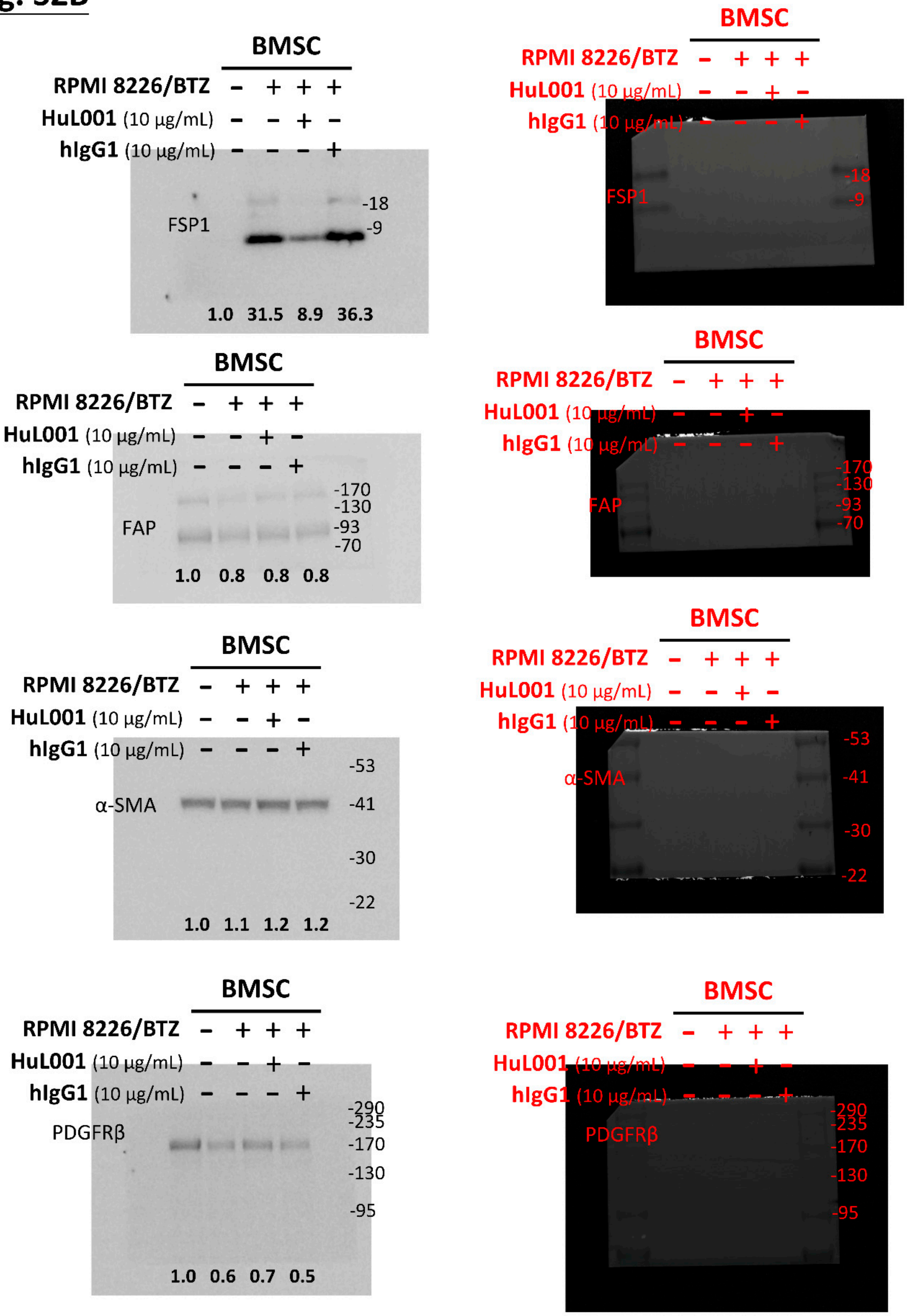

Figure S6 (continued 10).

Fig. S2B

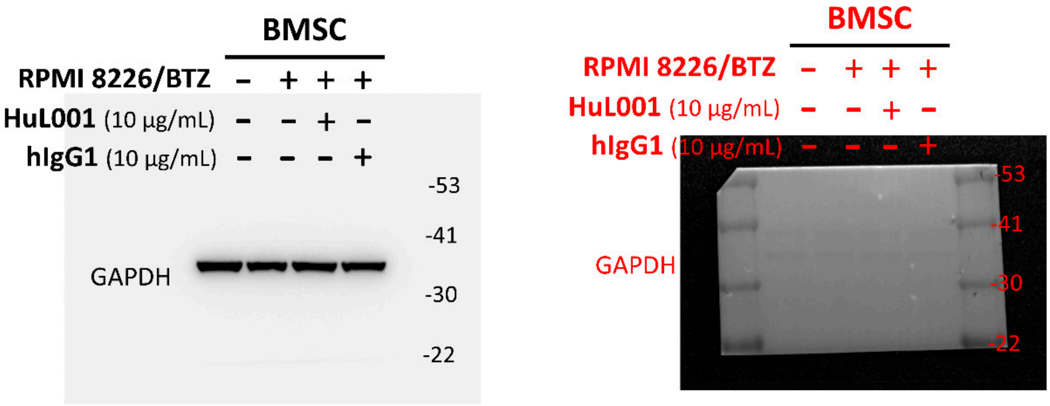

Figure S6 (continued 11).

Fig. S4A

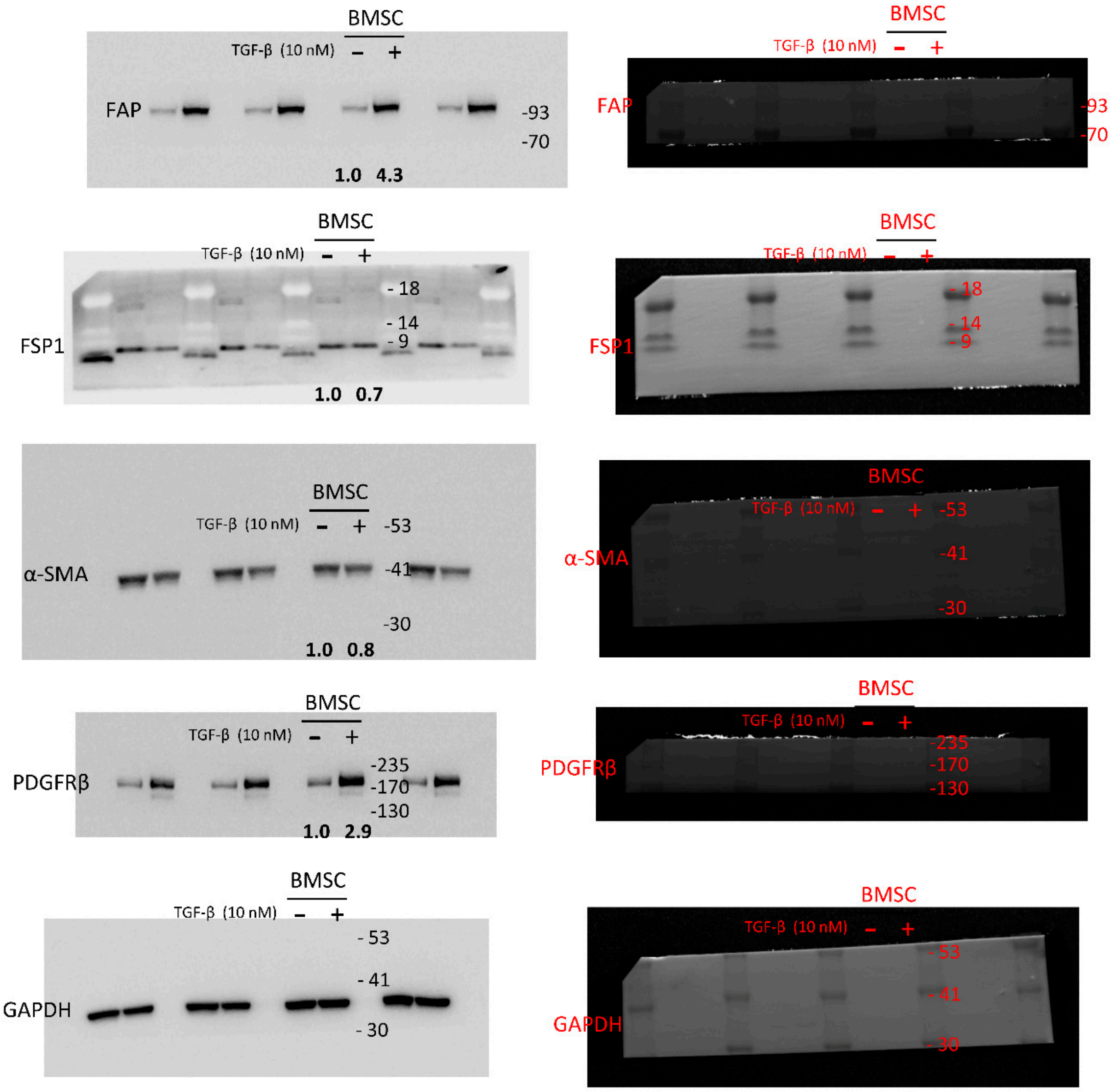

Figure S6 (continued 12).
